# Physical constraints determine the logic of bacterial promoter architectures

**DOI:** 10.1093/nar/gku078

**Published:** 2014-01-28

**Authors:** Daphne Ezer, Nicolae Radu Zabet, Boris Adryan

**Affiliations:** ^1^Cambridge Systems Biology Centre, University of Cambridge, Tennis Court Road, Cambridge CB2 1QR, UK and ^2^Department of Genetics, University of Cambridge, Downing Street, Cambridge CB2 3EH, UK

## Abstract

Site-specific transcription factors (TFs) bind to their target sites on the DNA, where they regulate the rate at which genes are transcribed. Bacterial TFs undergo facilitated diffusion (a combination of 3D diffusion around and 1D random walk on the DNA) when searching for their target sites. Using computer simulations of this search process, we show that the organization of the binding sites, in conjunction with TF copy number and binding site affinity, plays an important role in determining not only the steady state of promoter occupancy, but also the order at which TFs bind. These effects can be captured by facilitated diffusion-based models, but not by standard thermodynamics. We show that the spacing of binding sites encodes complex logic, which can be derived from combinations of three basic building blocks: switches, barriers and clusters, whose response alone and in higher orders of organization we characterize in detail. Effective promoter organizations are commonly found in the E. coli genome and are highly conserved between strains. This will allow studies of gene regulation at a previously unprecedented level of detail, where our framework can create testable hypothesis of promoter logic.

## INTRODUCTION

Bacterial promoters are often complex, containing many densely spaced and potentially overlapping transcription factor (TF) binding sites (1). The rate of gene expression depends on the promoter configuration (the specific combination of TFs that are bound to the promoter), and specific rules (‘logic’) may simply depend on the presence of two physically interacting TFs. Here, we propose that the dynamics of TF binding can influence promoter occupancy over time and therefore provide a time-dependent trigger that determines how TFs can depend on binding site spacing to influence gene expression.

One common method of identifying where TFs bind is to search a DNA sequence for TF binding site motifs, as specified by position weight matrices (PWMs) (2). Frequently, PWMs are used alongside statistical thermodynamic-based methods to incorporate additional properties influencing TF binding, such as TF concentration and spatial hindrance between TFs (3–14).

These thermodynamic ensemble models assume that the probability of a configuration occurring is directly correlated with the thermodynamic stability of that configuration, which is primarily influenced by the binding site affinities and protein abundances. However, some thermodynamically stable configurations may take a long time to form, thereby decreasing the likelihood that those configurations occur within the time frame of a cell cycle. To model promoter configuration without requiring strong assumptions about the presence of thermodynamic equilibrium, the kinetics underlying TF binding must be taken into account.

Both *in vitro* and *in vivo* studies have shown that TFs find their sites by facilitated diffusion (15–19); note that reference (19) provided strong evidence that TFs use facilitated diffusion as a translocation mechanism *in vivo*. This mechanism assumes that proteins do not home in on their target sites by 3D diffusion alone, but also take a random walk linearly along the DNA, in effect reducing the dimensionality of the search to find their binding sites more efficiently (20–24).

There have been many attempts to mathematically analyse the facilitated diffusion mechanism using analytical solutions (20,21,25–37). However, these mathematical approximations frequently assume a uniform affinity landscape and do not capture the stochastic behaviour of the system. We have previously established a stochastic simulation framework called GRiP (Gene Regulation in Prokaryotes) that can incorporate real affinity landscapes and therefore provides more accurate predictions of TF binding kinetics (23,38,39). *In vivo* single-molecule microscopy experiments have been used to measure various physical parameters in the facilitated diffusion process of the *Escherichia coli* TF *lacI*, including the average length of time *lacI* is bound to the DNA during its random walk, the average distance *lacI* traverses during its random walk and the proportion of time *lacI* is undergoing 1D diffusion versus 3D diffusion (18,19). We derive all of the kinetic parameters in our simulations from these measured experimental values (39); see Supplementary Table S1.

Transcriptional logic refers to the idea that the output—the expression level of a gene—depends on the specific combination of multiple inputs, the concentrations of TFs that regulate that gene. Typically, one considers the system to be in steady state, with the binding of the TFs to the promoter to be in quasi-equilibrium [e.g. (1,5,10)]. Here we extend this notion by proposing that the response to multiple inputs can also depend on the kinetics of TF binding, e.g. on the order by which the TFs bind to the promoter. In this context, we suggest that the spatial organization of the promoter encodes the logic of how TF concentration influences the promoter occupancy dynamics in biologically relevant time scales. Based on the facilitated diffusion model, we identified three basic functional units of diffusion-based transcriptional logic: (i) the switch (two overlapping TF binding sites), (ii) the barrier (two closely spaced, but non-overlapping TF binding sites) and (iii) the cluster (two closely spaced or overlapping binding sites for the same TF). Furthermore, we use the behaviours of these promoter building blocks to develop a semi-analytical model of the facilitated diffusion mechanism, which is significantly less resource-intensive than fully stochastic simulations and thus allows for genome-wide investigation. We then systematically describe the theoretical behaviour of these building blocks across possible concentrations and binding affinities and demonstrate that combining these building blocks can result in more sophisticated promoter behaviours. Finally, we show the distance between binding sites is highly conserved, thus supporting the idea that bacterial evolution may be partially driven by the physical constraints imposed by the TF search mechanism.

## MATERIALS AND METHODS

### GRiP simulations of promoter building blocks

We used GRiP to simulate the facilitated diffusion mechanism (38,39). GRiP models the diffusion of TFs implicitly via the Chemical Master Equation, as described in (40). Although other facilitated diffusion simulations incorporate the 3D structure of DNA (32,36,41–43), we do not because TF arrival times in *E. coli* are not significantly dependent on the 3D organization of DNA (44). The system was parameterized with values estimated from experimental data (39) and each simulation was run for 3000 s, approximately the *E. coli* cell cycle (45). We used the system-size reduction described in (46). The full list of parameters is listed in Supplementary Table S2.

### fastGRiP simulations of expanded parameter spaces and complex promoters

Analytical solutions of facilitated diffusion are faster than our GRiP simulations and can provide more insight into the mechanisms underlying the system, but they cannot incorporate real affinity landscapes. We developed a semi-analytical model (which we call fastGRiP) that uses mathematical approximations for the diffusion of TF molecules on non-specific DNA.

Our semi-analytical model uses a continuous time Markov chain, where each state represents a possible promoter configuration. Each transition represents the propensity of a single binding, unbinding or relocation reaction (in the case of a cluster). The reaction propensity is equal to 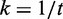, where *t* is the expected time for the reaction to occur.

The size of the Markov chain is 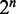, where *n* is the number of binding sites in the system. This means that the Markov chain grows rapidly with the number of binding sites and, thus, it becomes difficult to solve the system analytically (47), so we use the exact stochastic simulation algorithm (48,49), which generates a statistically correct trajectory through the Markov Chain (50).

### Binding event

The propensity of binding is calculated by an adaption of an equation described in (51)
(1)
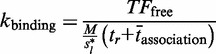

where 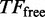 represents the number of unbound TF, *M* represents the length of the DNA segment that is being modelled, *t_r_* represents the time spent during a 1D random walk and 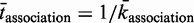 represents the time a single TF spends during 3D diffusion, where 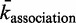 is the adjusted association rate to the DNA when assuming a smaller DNA segment; see (46).

When a TF binds randomly to the DNA, it has a 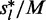 probability of landing within a sliding length of its binding site. The probability of binding can be expressed as a geometric distribution with expected value of 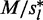 (the TF is expected to bind after 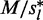 search attempts). Each search attempt takes 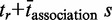: the time spent during the random walk plus the time spent undergoing 3D diffusion.

If TFs are spaced far apart, 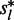 equals the sliding length, which is approximated by 90 bp based on experimental evidence for *lacI* (18). When a nearby binding site is occupied (the barrier case), 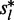 would represent the size of the reduced region from which a TF could find its binding site during a random walk. For instance, if there was a barrier 1 bp away from the binding site, 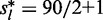. This approximation is supported by a recent study (52), which performed coarse-grained molecular dynamics simulations of TFs performing facilitated diffusion and showed that by increasing molecular crowding on the DNA (and, thus, the probability of a barrier forming in the neighbourhood of the binding site) leads to an increase in the number of 1D random walks required to locate the binding site.

When a TF has closely spaced binding sites (the cluster case), and one of the sites is already bound, 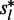 is the same as in the case of a barrier. Even when neither site is bound, the value of 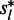 must be reduced because the TF will bind to the first site reached. In the case of two 20 bp length binding sites that are 1 bp away from each other, 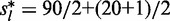.

A bound barrier or a bound/unbound cluster would result in a lower probability of a TF randomly landing within a sliding length of its binding site, so the propensity of binding is lower.

### Unbinding event

The propensity of unbinding events can be written as
(2)
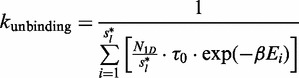

where 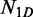 is the number of sliding events in the random walk (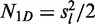) (53), 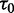 is the amount of time spent at the strongest target site and 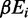 represents the binding energy at position *i* in the region over where the random walk takes place (54).

The size of the region over which the random walk 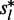 is calculated as before, except that 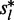 does not need to be adjusted in the case of unbound clusters, because the neighbouring sites do not restrict the random walk.

When there are barriers, the TF will visit its preferred binding site more often than usual because its random walk is restricted, thereby increasing the time the TF spends bound. If the second TF in a cluster is unbound, the first TF will sample both binding sites during its random walk and will therefore remain bound much longer.

### Relocation event

The expected time that a molecule moves by 1D random walk from one site to a nearby one, located *d* nucleotides away, is equal to the expected time of the random walk between the two sites:
(3)
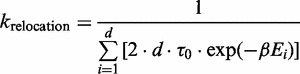

where *d* is the distance between the two sites.

### Assumptions of fastGRiP

A main assumption of fastGRiP is that TF binding to non-specific sites can be approximated by an analytical solution. We see that fastGRiP and GRiP provide statistically equivalent outcomes, indicating that this assumption likely holds.

Other factors that are not simulated explicitly in GRiP might influence protein localization. For instance, fastGRiP does not directly take into account TF-TF interactions, but some of the behaviours of TF-TF interactions can be indirectly included. TFs may influence the binding dynamics via dimerization or recruitment. If two TFs first dimerize and then bind to the DNA, they can be treated as a single TF in the model, but if two TFs individually bind and then dimerize on the DNA (or if one TF recruits a neighbouring TF by influencing the binding affinity of the neighbouring site), fastGRiP will not be able to model this behaviour yet, and one should use a comprehensive computational model to simulate the facilitated diffusion mechanism (such as GRiP).

In addition, TFs may provide steric hindrance to the left and the right of the binding site, and these values may be estimated from DNAse I and MNase footprinting (55).

Finally, our semi-analytical model (fastGRiP) is just an approximation of a more comprehensive model that considers facilitated diffusion (GRiP) and, thus, it may not capture some of the noise that is generated by the non-uniform landscape and by non-cognate TF molecules.

## RESULTS

### Promoter logic building blocks

**Figure 1.**
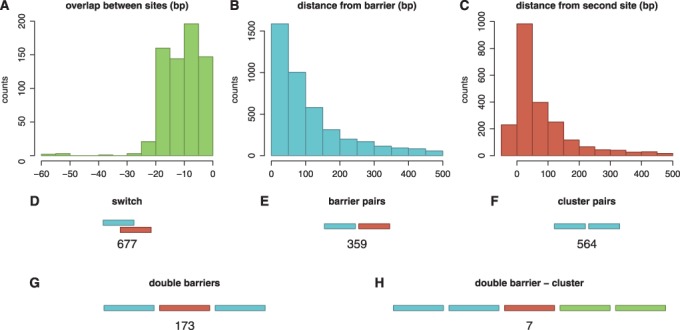
Distribution of promoter architectures in *E. coli*. We considered the binding sites in *E. coli* K-12, which were listed in RegulonDB (58). (**A–C**) plot histograms of the overlap or the distance between two sites that form a (A) switch, (B) barrier and (C) cluster. Next, we counted the number of (**D**) switches, (**E**) barriers and (**F**) clusters and found that these building blocks are frequently encountered in *E. coli* K-12 genome. For the barrier and cluster pairs, we consider binding sites that are <10 bp apart. In (**G**) and (**H**), we presented some examples of complex promoters: (G) double barrier and (H) double barrier cluster.

Facilitated diffusion influences the rate at which promoter configurations form by affecting the association and dissociation rates of TFs to/from their target sites. It has been shown both theoretically [44,56) and experimentally (19) that a TF bound to a strong binding site can form an obstacle that slows the rate of binding of a neighbouring TF. Other studies have suggested that multiple adjacent binding sites for the same TF might enhance TF binding (41) and increase gene expression (57). These experiments and simulations suggest that the spacing of TFs help encode transcriptional logic. Here we consider three promoter building blocks in which the spacing between TF binding sites influences the dynamics of TF binding: switches, barriers and clusters. These three components are found frequently throughout the *E. coli* genome; see Figure 1D–F.

To investigate the binding of TFs to these three building blocks, we simulated the process by which TFs search for their binding sites using the stochastic simulation framework GRiP (23,38,39,46). The details of the model and parameters are listed in the ‘Materials and Methods’ section and in Supplementary Tables S1, S2 and S3. Please note that some additional results were obtained with an approximation of GRiP called fastGRiP, as detailed later this paper (‘Approximating GRiP with fastGRiP’ section) and in the ‘Materials and Methods’ section.

### Switches

Many TFs in the *E. coli* genome have overlapping binding sites; Figure 1D. If two or more TFs have overlapping binding sites, only one of the TFs can bind to that position at a time, resulting in a ‘switch’-like behaviour.

**Figure 2.**
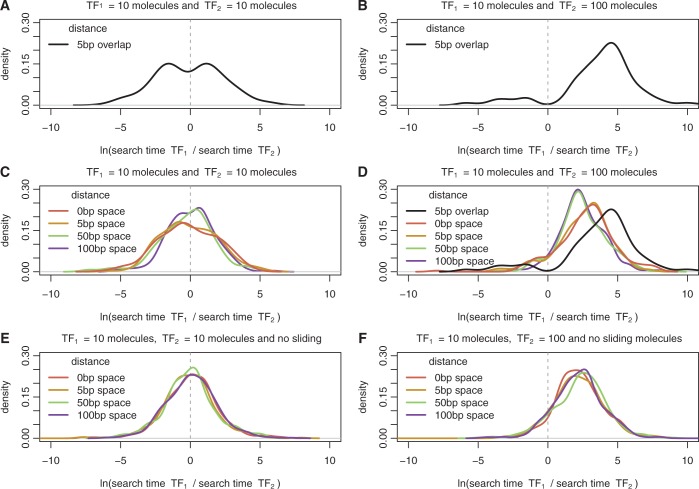
Ratio of TF arrival times for switches and barriers. Here we show the density plot of the difference in arrival to the two target sites for (**A**) and (**B**) switches (overlapping sites by 5 bp) and (**C**), (**D**), (**E**) and (**F**) barriers (distances 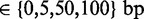). We considered an overlap of 5 bp because it is the average overlap between two adjacent binding sites; see Figure 1A. We simulated facilitated diffusion in (A–D), but only 3D diffusion in (E) and (F). The set of parameters for the TFs performing facilitated diffusion are listed in Supplementary Table S2, while the set of parameters for the system TFs performing only 3D diffusion are listed in Supplementary Table S3. In (A), (C) and (E) the two TF species have the same abundance (10 molecules), while in (B), (D) and (F) the second TF is 10 times more abundant than the first TF (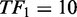 and 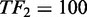). Note that in (D) to emphasize the dependence of the arrival time on the distance we also plot the case of overlapping binding sites.

Here, we simulated with GRiP a switch system formed of two overlapping binding sites with two TFs (*TF*_1_ and *TF*_2_) and we measured the ratio of their respective times to first binding. Figure 2A shows that the log ratio of the arrival times [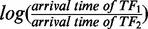] display a bimodal distribution, with a *P*-value of 0.037 when performing the dip test (59,60), where each mode of the distribution represents the case of a different TF arriving first.

A particular TF’s probability of binding in this competitive environment is also influenced by the differing concentrations and binding affinities of the alternative TFs. TFs with higher concentrations find their binding sites faster than TFs with comparatively lower concentrations (due to a higher number of molecules searching for the binding site); see Figure 2B. Similarly, a TF with a higher binding affinity would remain bound to the DNA for a longer period, thereby preventing other TFs from attaching to its site; see Supplementary Figure S1. These results are also valid in the case of non-uniform affinity landscape; see Supplementary Figure S2.

### Barriers

Next, we investigated the influence of adjacent, but non-overlapping binding sites on promoter configuration. Previously, it has been shown that the rate of TF binding can be slowed by decreasing the distance between adjacent but non-overlapping TF binding sites (19,56). We refer to this interference as the ‘barrier effect’.

Even though properties such as DNA bending and electrostatic interactions between TFs could help explain these results, (19) has demonstrated that facilitated diffusion is sufficient to explain the observed barrier effect in the case of a barrier containing *LacI* and *TetR* binding sites. Previous research found that the TF molecules slide along the DNA maintaining a specific orientation with respect to the DNA (following a helical path) (61). This supports the idea that, in a facilitated diffusion-based model, a TF can find its binding site by 1D diffusion from two directions (by diffusing to the binding site from an upstream or downstream direction). When one of the TFs in a barrier is bound, the other TF can only find its binding site by 1D diffusion from one of these directions, thereby making its arrival less probable and increasing the average time to binding.

Our results support the work of (19); see Figure 2D. In particular, non-overlapping binding sites that are within half of the sliding length, the binding of the least abundant TFs is slowed down, but not as much as in the case of overlapping binding sites. When the binding sites are far apart (further than half of the sliding length), the facilitated diffusion mechanism does not significantly influence the rate of TF binding.

To demonstrate that this result is consequence of facilitated diffusion, we compared our standard GRiP simulations with those that only included 3D diffusion. When our model only enabled TFs to find their binding sites via 3D diffusion (no 1D diffusion), the distance between the binding sites had no impact on the TF arrival times; compare Figure 2C with D. This confirms that the barrier effect was a direct consequence of 1D diffusion along the DNA in our simulations.

Barriers do not significantly affect the total amount of time the TFs spend bound to their binding sites across a cell cycle (the ‘total occupancy’); see Supplementary Figure S3. This is the result of two opposing effects: on one hand, the barrier effect increases the average time it takes for a TF to reach its binding site as shown in Figure 2D (19,44), while, on the other hand, once the TF is bound, it stays bound longer by restricting the ability of TF molecules to diffuse away from their sites (44,62). Based on the physics equations we derived for fastGRiP (see ‘Materials and Methods’ section), we can demonstrate that although the total occupancy is not significantly different, the rate of TF binding ‘and’ unbinding are reduced in the barrier case; see Supplementary Figure S4.

### Clusters

Unlike barriers, whose binding sites are for different TFs, clusters contain multiple binding sites for the same TF. Therefore, a suitable TF can bind at any site in a cluster. Bacterial cells frequently have multiple copies of the same binding site clustered together; see Figure 1F and (1). Experiments have shown that TF binding site clustering can enhance gene expression (57).

If we consider the facilitated diffusion mechanism, clusters display two opposing behaviours, namely (i) a TF can slide back and forth between the two nearby binding sites (thus increasing occupancy within that region) and (ii) a bound TF can act as a barrier to the other binding site and a neighbouring empty binding site can also act as a trap, as the TF will attach to the first binding site it reaches, also slowing the rate of binding (thus slowing the rate of binding of other molecules to the second site); see Supplementary Figure S5.

**Figure 3.**
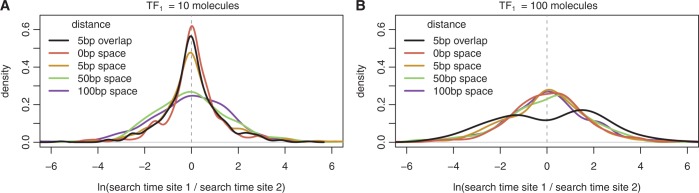
Ratio of TF arrival times in clusters. We show the density plot of the difference in arrival to the two binding sites (distance between sites 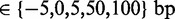) of the same TF, *TF*_1_. In (**A**), *TF*_1_ has low abundance (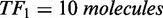), and in (**B**), *TF*_1_ has high abundance (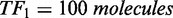).

The balance between these two opposing behaviours depends on the concentration and binding affinity of the binding sites. For instance, clusters enhance TF binding rates at low concentrations (Figure 3A). However, clusters do not enhance binding rates when the concentration of the TF is sufficiently high (Figure 3B). In fact, we see an increased degree of bimodality in overlapping bindings sites in a cluster than in a standard switch (with a *P*-value of 0.025 when performing the dip test in the cluster case, as opposed to a *P*-value of 0.037 in the switch case).

### Complex configurations

Complex promoters are common in the *E. coli* genome: there are 195 sets of three or more binding sites that are all separated by <100 bp; see Figure 1G. These complex promoters have diverse structures in the sense that every possible combination of switches, barriers and clusters that could be constructed from 3, 4 or 5 binding sites will be found in the *E. coli* genome; Supplementary Figure S6 introduces a nomenclature that we developed to systematically catalogue promoter architectures. An example of switching, barrier and clustered promoters (and combinations thereof) with four different binding sites is shown in Supplementary Figure S7. The entire data set of all promoter architecture classifications is accessible at http://logic.sysbiol.cam.ac.uk/fgrip/db; as introduced in Supplementary Figure S8.

A particular promoter architecture may be enriched in the genome because of evolutionary selection for a certain functional role or as a byproduct of the process by which mutations occur (63,64). For instance, clusters can arise from local DNA sequence duplication (65), a common mutation event. Therefore, similar binding sites tend to be co-located on the genome rather than being interspersed with unrelated motifs (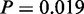, chi-squared test).

Interestingly, there is an enrichment for alternating switch-barrier architectures as compared with architectures with the same ratio of switches and barriers (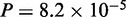, chi-squared test; see Supplementary Figure S8), and this architecture is enriched for genes activated by nitrite/nitrate (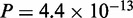, binomial probability distribution), suggesting that this architecture might play an important role. To understand possible functional roles of complex promoter architectures, we simulated the TF search process of a number of these complex promoters.

### Approximating GRiP with fastGRiP

Fully stochastic simulations (such as GRiP) are too computationally intensive to simulate complex systems. Analytical solutions of facilitated diffusion are faster and provide more insight into the mechanisms underlying the system, but they cannot incorporate real affinity landscapes. Therefore, we developed a semi-analytical model (fastGRiP), which is based on the behaviour of the three building blocks (switches, barriers and clusters). fastGRiP uses a continuous time Markov chain, where each state represents a possible promoter configuration and each transition represents a single binding, unbinding or relocation event (the case of a TF jumping between two adjacent TF binding sites in a cluster). The full description of fastGRiP and the associated equations are presented in ‘Materials and Methods’ section. It is compared with GRiP in Supplementary Figure S9 and the runtime is analysed in Supplementary Figure S10.

### Two-sided barriers

The first complex promoter that we investigated is the *ABA* pattern, which has two identical sites that surround another site. Each adjacent pair of TF binding sites forms a barrier, and the two binding sites of the same type can form a cluster if the binding sites are close enough to one another; see Figure 1G.

In the building block section, we described how a bound TF slows the rate of binding to an adjacent site via the barrier effect. We wished to see how much this barrier effect would be amplified if barriers surrounded a TF binding site on both sides. Therefore, we focused our analysis on how the ABA pattern affected the ability of all three TFs to be bound at once, what we call the ‘AND configuration’.

**Figure 4.**
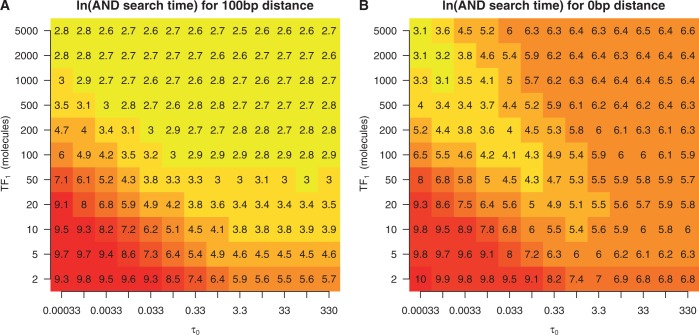
Heatmap of the ln of the first time all three sites are occupied in double sided barriers. We considered the promoter configuration ABA, where A is the target site of *TF*_1_ and B is the target site of *TF*_2_. The system consists of 10 molecules of *TF*_2_ with a binding affinity scaling parameter of 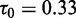; see ‘Materials and Methods’ section. The distance between adjacent binding sites is (**A**) 100 bp and (**B**) 0 bp. We vary the abundance and DNA binding affinity of *TF*_1_.

When the binding sites were 100 bp apart (far enough ‘not’ to be influenced by the barrier effect), the simulations predicted that increasing the concentration and binding affinity of the TF binding to the outer binding sites would increase the likelihood that all three sites are occupied simultaneously (the ‘AND configuration’); see Figure 4A. Because the binding sites are far enough away as to not be influenced by the barrier effect, these results are consistent with the expected outcome of a thermodynamic ensemble model.

In contrast, when the binding sites are close together (0 bp), we observed a behaviour that cannot be explained within the thermodynamic framework. More specifically, at high concentrations and binding affinities, the ‘AND configuration’ forms slowly because the outer two binding sites are frequently bound, restricting the binding of the central binding site (through the barrier effect). Furthermore, when both the concentration and binding affinity of the outer sites are low, the ‘AND configuration’ forms slowly because the outer sites are unlikely to be occupied concurrently. The case in which the ‘AND configuration’ forms fastest is when the outer binding sites have a low binding affinity and high abundance; see Figure 4B.

These results also illustrate the magnitude to which the arrival times of TFs can be affected by facilitated diffusion. For instance, when the TFs that bind to the outer two binding sites had an abundance of two molecules and a 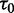 of 0.33, the ‘AND configuration’ formed in ∼1600 s (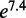) in the case of far away TF binding sites, and in ∼3600 s (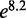) in the case of closely spaced TF binding sites. Therefore, on average, the barrier effect delayed the formation of the AND configuration by 2000 s in this scenario. Note that, while in the former case, the average time to reach the AND configuration is half of the *E. coli* cell cycle (the cell cycle is ∼3000 s), in the latter case, this time is longer than the average length of the cell cycle. This example shows that the spacing between binding sites can influence the timing of TF binding events at time scales, which would be biologically relevant. In fact, we found that the percentage of simulations where the AND configuration is reached within half of cell cycle is dependent not only on the number of binding sites in the promoter but also on the distance between the sites; see Supplementary Figure S11.

Next, we investigated whether this model could provide insight into genome organization. In the *E. coli* genome, there are several hundred triplets of closely spaced binding sites, including *pdhR*, *dpiBA* and *moeAB*. Among these triplets that are <50 bp apart, the outer binding sites are more likely to have lower binding affinities than the central binding site (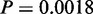, Chi-squared test; see Supplementary Figure S12). In addition, there is strong anti-correlation (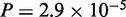, Fisher exact test) between having stronger binding sites and having higher TF concentrations, as measured by APEX (66), in the TFs binding to the outer binding sites compared with the TF binding to the inner binding site; see Supplementary Figure S12. This suggests that the organization of promoters in *E. coli* may be optimized for having multiple TFs bound at once (instead of two or one) or optimized for allowing binding to the middle binding site. The ‘AND configuration’ has been shown to play an important role in *E. coli*. Cox *et al.* (67) constructed 288 synthetic promoters in *E. coli* and found that the preferred transcriptional mode between three adjacent sites is the AND logic.

### Double barrier cluster

Next, we considered the case of a site being flanked by two identical clusters (the *AABAA* pattern) with 0 bp between each adjacent pair of TFs. We wished to determine whether combining clusters and barriers would produce a promoter logic pattern that could not be explained by the behaviour of clusters or barriers alone. We compared the behaviour of the *AABAA* pattern to similar scenarios containing only barriers (five different adjacent TFs, an *ABCDE* pattern) and only clusters (two pairs of clusters separated from a central TF by 100 bp, an *AA-B-AA* pattern).

**Figure 5.**
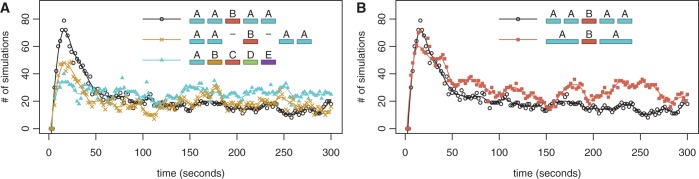
Impulse behaviour of AABAA. We show the number of simulations (out of 400) have only the central binding site bound, over time, for the AABAA configuration compared with (**A**) AA-B-AA and ABCDE and (**B**) ABA. For example, in the AABAA scenario, the y-axis represents the number of simulations out of the 400 in which the B site is bound and none of the A sites are bound. Note that we start the simulation with ‘naked’ DNA (no TFs bound).

In the AABAA scenario, when we graph the number of simulations in which only the central TF is bound, over time (starting from DNA that has no TFs bound), we observe an impulse behaviour—a short period in which there is a higher probability of this configuration occurring than observed at equilibrium; see Figure 5A. Alternatively, the graph can also be read as the number of cells in a population in which only the central TF is bound after x seconds. If we were to consider *TF_B_* to be an activator and *TF_A_* to be a repressor, an impulse could possibly result in a short burst of gene expression.

The impulse behaviour is influenced by (i) how often the central TF binds first (ii) how long this configuration lasts before other TFs come and bind to the DNA. In the AABAA configuration, *TF_B_* (binding to site B) has a relatively high rate of binding because the A binding sites (the clusters) will act as obstacles to one another because a *TF_A_* will bind to the first A binding site it encounters. This configuration will also remain a relatively long time because the central bound TF (*TF_B_*) acts as a barrier that slows the rate of binding of the other TFs.

When there are no clusters (ABCDE), all the TFs have an equal probability of binding first. When there are no barriers (AA-B-AA), *TF_B_* cannot act as a barrier to slow the binding of other TFs, so alternate configurations are more rapidly assumed because the binding of other TFs is not obstructed. Therefore, in both the ABCDE and AA-B-AA configurations the size of the impulse is reduced. This illustrates that the combination of barriers and clusters can result in different responses than each component independently; see Supplementary Figure S13.

We wondered whether the *AABAA* pattern acted similarly to the double barrier (*ABA*) described in the previous section. The presence of a cluster would increase the time a TF is bound to the DNA and the rate of TF binding; however, we were curious if additional factors beyond these two also contributed to the logic of the AABAA promoter. We simulated a double barrier *ABA* pattern with the outer two TFs having twice the length and twice the binding strength. This scenario displayed similar impulse behaviour, but the results displayed more stochasticity than the *AABAA* pattern (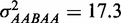 and 
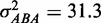
, while the Fano factor for the AABAA configuration is 1.02 and for the ABA configuration is 1.15; F-test: 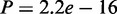); see Figure 5B. This is supported by recent single cell imaging study that suggests that increasing the strength of a binding site could increase transcriptional noise (68).

These results indicate that the combination of clusters and barriers can create qualitatively different behaviours than their individual components and that promoter organizations influence the stability of specific promoter configurations.

### Evolution of complex promoters in *E. coli*

Because the behaviours of barriers and clusters depends on the spacing of binding sites, one would expect there to be evolutionary selection pressure keeping binding site spacing conserved during the evolution of promoters in *E. coli*.

We compared the insertion–deletion (indel) rates and the single base pair substitution rates of different regions of promoters. The evolutionary events were parsed into the following categories: between transcription start site (TSS) and first binding site, within binding sites, between closely spaced binding sites, between binding sites farther than 100 bp apart and between the last binding site and the termination sequence. The indel rates and base pair substitution rates were calculated in a pair-wise fashion between *E. coli* K-12 (the main *E. coli* reference genome) and the other five NCBI-designated reference *E. coli* genomes (O157:H7, IAI39, UMN026, O83:H1 and O104:H4). Note that we control for the different DNA sequence lengths in each category because our mutation rates are defined as 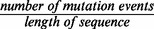.

**Figure 6.**
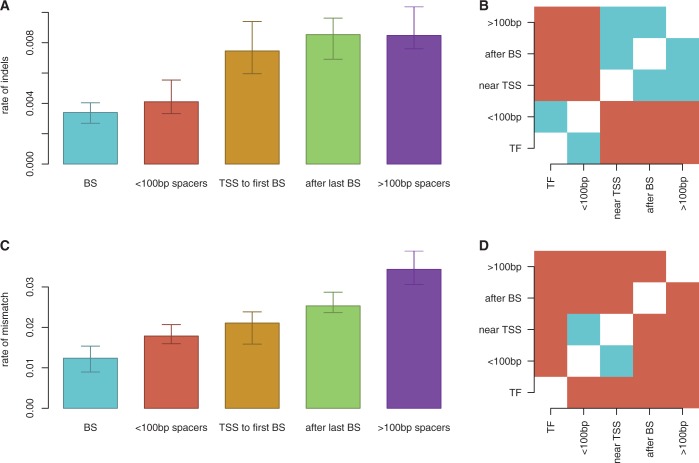
Differentialevolution of promoter components in *E. coli*. We compared (**A**) and (**B**) indel rates and (**C**) and (**D**) SNP rates between *E. coli* K-12 and other reference strains across different promoter regions. (B) and (D) display results of a paired-T-test with Holm corrections; significantly different promoter regions are red and insignificant pairs are blue (threshold: 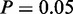). We considered the following five cases: (A) within binding sites (BSs), (B) between closely spaced binding sites (<100 bp spacers), (C) between binding sites farther than 100 bp (>100 bp spacers), (D) between TSS and first binding site (TSS to first BS) and (**E**) between the last binding site and the termination sequence (after last BS).

Figure 6 shows that regions between closely spaced TFs (<100 bp apart) had similar indel rates as TF binding sites, but had significantly higher rates of single base pair substitutions. In contrast, regions between distant TF binding sites (>100 bp apart, and therefore not influenced by facilitated diffusion) had much higher rates of indels and mismatches. These results indicate that regions between closely spaced TF binding sites are conserved in terms of length, but not as highly conserved in terms of sequence. We see that across all closely spaced TF binding sites in all six strains, only two TF binding sites change their relative distance by >1 bp. This suggests that there may be evolutionary selection pressure to keep the distances between TF binding sites conserved. Although this evolutionary analysis does not provide direct evidence that facilitated diffusion plays a functional role, these results are compatible with our proposition that TF binding site spacing may have a facilitated-diffusion driven functional role.

Nevertheless, binding site spacing may also be conserved for other reasons. For instance, the distance between adjacent binding sites within promoters are conserved to preserve the distance between the binding sites and the TSS (to conserve the effect of the binding site on the gene regulation). To investigate this assumption, we also looked at the conservation of the distance between the first binding site in the *cis-*regulatory region and the TSS and found that these regions are not conserved in both distance and sequence; see Figure 6. This indicates that the conservation of the distance between binding sites is not influenced by the distance between the binding sites and the TSS.

Additionally, TFs that display direct interactions with other TFs or act via DNA-bending may have certain binding site spacing requirements. If this was the primary reason for the conservation of TF spacing, the spacing of TF binding sites that act via these mechanisms would be more conserved than those that do not. However, we see that the spacing between ‘all’ closely packed TFs are significantly conserved, suggesting that either ‘most’ closely spaced TF binding site pairs participate in TF–TF interaction or other mechanisms (such as facilitated diffusion) could also influence the selection pressure acting on preserving the distance between binding sites.

## DISCUSSION

The prediction of TF binding to promoters has received significant attention in the literature, as it is the first step towards developing mechanistic models of gene expression. Transcriptional logic is often assumed to be independent of the spacing of TF binding sites and only associated with which TFs can bind to the promoter. The underlying principle behind this assumption is that the cell operates as a well-stirred reactor, which can lead to misleading results because of rapid TF-DNA rebindings (40).

Alternatively, the binding of TFs has been predicted by scanning the DNA for a PWM and then calculating the probability of binding using a statistical thermodynamic framework to take into account TF concentration (3–5,7–9) and steric hindrance on the DNA (6,10–12,14). However, these models assume that TFs are bound at thermodynamic equilibrium, even though thermodynamic equilibrium might not be reached in the time frame of a cell cycle.

Within a facilitated diffusion context, the distance between TF binding sites on the DNA could potentially encode for transcriptional logic in a way that the classical thermodynamic models cannot capture (56). In fact, experimental studies have shown that when binding site spacing is manipulated, the occupancy of the site is affected (19) and this can even influence transcription (57,67,69). In this article, we present a theoretical explanation of how binding site spacing could encode facilitated diffusion-based transcriptional logic.

### Combining promoter logic building blocks to form complex promoters

We identified three examples of how spacing between binding sites can influence the dynamics of TF binding, namely, switches, barriers and clusters.

Previous studies have suggested that switches influence transcriptional logic of prokaryotic promoters (1); see Figure 2A and B). *In vivo* experimental studies show that binding site occupancy depends on the distances between TF binding sites, due to the ‘barrier effect’ (19) and this change in occupancy can affect gene transcription (57,67,69); see Figure 2C–F. Finally, we found that under certain TF abundances and affinities, when two identical sites are close (clusters) the difference in association rates to the two sites is reduced, as suggested in (70); see Figure 3. These three building blocks are found frequently in the *E. coli* genome; see Figure 1D–F.

Although others have suggested that co-localization of TF binding sites can influence the dynamics of TF binding, none of these studies have analysed complex promoters that combine barriers, switches and clusters, which can encode complex behaviours. Describing promoters in terms of these building blocks gives us a language to help classify complex promoters by their structures and look for patterns in promoter organization. Our analysis also indicates that taking into account the distribution of promoter building blocks may be a useful lens for evaluating the evolution of promoters.

### Complex promoters

Two of the complex configurations that we studied in depth were the double barrier (an ABA configuration) and the double barrier cluster (an AABAA configuration). In the double barrier scenario, we observed that the time for all TFs to find their binding sites depended on the concentration and binding affinity of the TFs. The promoter organization was optimized for having all three TFs bound at once when the outer TFs had higher concentration and lower binding affinity than the central TF; see Figure 4. Interestingly, this organization was significantly enriched within the *E. coli* genome.

In the double barrier cluster scenario, the promoter configuration can display a temporal impulse in the occupancy of the middle site. A similar type of response can also be produced by an incoherent feed-forward loop in the gene regulatory network (71). On one hand, the advantage of having the impulse response encoded in the occupancy of the promoter (and not in the gene regulatory network) is the fact that the response is faster and the metabolic cost is lower (the gene expression process is both slow and metabolically expensive for the cell) (72). On the other hand, the disadvantage of having the impulse response encoded in the occupancy of the promoter is that it becomes difficult to sustain the impulse for longer time intervals and this is where the gene regulatory network overcomes the limitation of the promoter occupancy. Hence, depending on the system requirements, i.e. biological context, the temporal impulse response can be encoded in the promoter configuration (faster response) or in the gene regulatory network (longer impulse).

### Facilitated diffusion as a lens for interpreting experimental data

Frequently there is an assumption that two TFs with correlated binding behaviours must interact directly (1,10,13). Here, we show that TFs can influence the binding of neighbouring sites without direct interaction. We agree that protein–protein interactions are important for determining transcriptional logic (73,74), and in many cases OR, XOR and AND logic could be primarily encoded in these interactions; however, screens for these interactions should consider a facilitated diffusion based model as their null hypothesis.

For example, Cheng *et al.* (74) used a statistical thermodynamics model to analyse ChIP data and identified that the data are best explained when including blocking of binding (antagonistic effects) even in the case when the sites do not overlap. Interestingly, they found a bias in the distance between the non-overlapping sites of up to 30 bp, which suggest a possible barrier effect being involved. We are not claiming that the facilitated diffusion is the only possible explanation for the observed behaviour, but rather that this might be one possible explanation for the observed results.

The possible functional role of TF spacing also opens up interesting questions in an evolutionary context: are the locations of TF binding sites influenced by the physics of diffusion? We compared the indel rates in six *E. coli* strains (K12, O157:H7, IAI39, UMN026, O83:H1 and O104:H4) and our results showed high conservation of the spacing between binding sites for spaces <100 bp (similar with the conservation of the binding sites themselves); see Figure 6. In contrast, the DNA sequence of the spaces between the binding sites is not conserved and neither is the distance between the binding sites and the TSS. Put together, these results suggest that the evolution of bacterial systems might be influenced by the facilitated diffusion mechanism.

### Computational tool

To aid biologists in analysing complex promoter behaviours, we provide a semi-analytical model (called fastGRiP) through an intuitive web interface (http://logic.sysbiol.cam.ac.uk/fgrip/; also see Supplementary Figure S14), which leads to only negligible deviations from the full model of facilitated diffusion (GRiP). It is significantly faster than GRiP and allows investigations of complex promoters under a wider set of parameters within short simulation times; see Supplementary Figure S10. Furthermore, our complete classification of *E. coli* promoters can be browsed at http://logic.sysbiol.cam.ac.uk/fgrip/db, with the option to download the data set for further analysis.

### Testing the proposed model with experiments

Our model predictions may be tested by constructing specific synthetic promoters and measuring TF binding kinetics (e.g. via *in vivo* single molecule microscopy experiments for low abundance TFs) (18,19) and gene expression (via quantitative polymerase chain reaction or luciferase assays). The parameters that one would wish to manipulate in these experiments include (i) the distance between binding sites (varied between 0 and 100 bp), by synthesizing different promoter sequences, (ii) the abundance of the TF, by using an inducible promoter to control the expression of the TF and (iii) the binding affinity of the TF to its binding site, which can be somewhat controlled by manipulating the DNA sequence of the binding motif or adjusting the salt concentration. Note that TF abundance and binding affinity can only be roughly adjusted, so only qualitative comparisons can be made.

For instance, to test whether the double barrier cluster scenario (AABAA) can generate a noticeable impulse behaviour in terms of gene expression, one needs to synthesize a promoter where the TF that binds to the middle site is an activator (e.g. CRP) and the TFs that bind to the surrounding clusters are repressors (e.g. lacI). Both TFs must be inducible, so that they can both be turned on at similar times, and a luciferase assay could be used to measure gene expression over time. We expect that the double barrier cluster scenario generates an impulse in gene expression, but that this would not be the case if the clusters are far apart from the central TF.

Biophysicists have studied facilitated diffusion for >30 years, but the focus has been on understanding the fundamental properties of this mechanism. Here we demonstrate that facilitated diffusion could influence the transcriptional logic of common *E. coli* promoter architectures and that these architectures are highly conserved between strains. We provide a framework, in terms of switches, barriers and clusters, for classifying promoter architectures by their facilitated-diffusion–based transcriptional logic and provide a web service to allow biologists to easily analyse the TF binding dynamics of bacterial promoters. We hope that this is a first step towards bridging between the facilitated diffusion and gene regulation research communities.

## SUPPLEMENTARY DATA

Supplementary Data are available at NAR Online.

## FUNDING

Marshall Scholarship (to D.E.); Medical Research Council Bioinformatics Training Fellowship [G1002110 to N.R.Z.]; Royal Society University Research Fellowship (to B.A.). Funding for open access charge: The University of Cambridge.

*Conflict of interest statement*. None declared.

## Supplementary Material

Supplementary Data

## References

[1] Hermsen R, Tans S, ten Wolde PR (2006). Transcriptional regulation by competing transcription factor modules. PLoS Comput. Biol..

[2] Stormo GD (2000). DNA binding sites: representation and discovery. Bioinformatics.

[3] Ackers GK, Johnson AD, Shea MA (1982). Quantitative model for gene regulation by lambda phage repressor. Proc. Natl Acad. Sci. USA.

[4] Djordjevic M, Sengupta AM, Shraiman BI (2003). A biophysical approach to transcription factor binding site discovery. Genome Res..

[5] Bintu L, Buchler NE, Garcia HG, Gerland U, Hwa T, Kondev J, Phillips R (2005). Transcriptional regulation by the numbers: models. Curr. Opin. Genet. Dev..

[6] Foat BC, Morozov AV, Bussemaker HJ (2006). Statistical mechanical modeling of genome-wide transcription factor occupancy data by MatrixREDUCE. Bioinformatics.

[7] Roider HG, Kanhere A, Manke T, Vingron M (2007). Predicting transcription factor affinities to DNA from a biophysical model. Bioinformatics.

[8] Chu D, Zabet NR, Mitavskiy B (2009). Models of transcription factor binding: sensitivity of activation functions to model assumptions. J. Theor. Biol..

[9] Zhao Y, Granas D, Stormo GD (2009). Inferring binding energies from selected binding sites. PLoS Comput. Biol..

[10] Raveh-Sadka T, Levo M, Segal E (2009). Incorporating nucleosomes into thermodynamic models of transcription regulation. Genome Res..

[11] Wasson T, Hartemink AJ (2009). An ensemble model of competitive multi-factor binding of the genome. Genome Res..

[12] Hoffman MM, Birney E (2010). An effective model for natural selection in promoters. Genome Res..

[13] Kaplan T, Li XY, Sabo PJ, Thomas S, Stamatoyannopoulos JA, Biggin MD, Eisen MB (2011). Quantitative models of the mechanisms that control genome-wide patterns of transcription factor binding during early drosophila development. PLoS Genet..

[14] Simicevic J, Schmid AW, Gilardoni PA, Zoller B, Raghav SK, Krier I, Gubelmann C, Lisacek F, Naef F, Moniatte M (2013). Absolute quantification of transcription factors during cellular differentiation using multiplexed targeted proteomics. Nat. Methods.

[15] Riggs AD, Bourgeois S, Cohn M (1970). The lac represser-operator interaction: III. Kinetic studies. J. Mol. Biol..

[16] Kabata H, Kurosawa O, IArai MW, Margarson S, Glass R, Shimamoto N (1993). Visualization of single molecules of RNA polymerase sliding along DNA. Science.

[17] Blainey PC, van Oijen AM, Banerjee A, Verdine GL, Xie XS (2006). A base-excision DNA-repair protein finds intrahelical lesion bases by fast sliding in contact with DNA. Proc. Natl Acad. Sci. USA.

[18] Elf J, Li GW, Xie XS (2007). Probing transcription factor dynamics at the single-molecule level in a living cell. Science.

[19] Hammar P, Leroy P, Mahmutovic A, Marklund EG, Berg OG, Elf J (2012). The lac repressor displays facilitated diffusion in living cells. Science.

[20] Berg OG, Winter RB, von Hippel PH (1981). Diffusion-driven mechanisms of protein translocation on nucleic acids. 1. Models and theory. Biochemistry.

[21] Halford SE, Marko JF (2004). How do site-specific DNA-binding proteins find their targets?. Nucleic Acids Res..

[22] Mirny L, Slutsky M, Wunderlich Z, Tafvizi A, Leith J, Kosmrlj A (2009). How a protein searches for its site on DNA: the mechanism of facilitated diffusion. J. Phy. A Math. Theor..

[23] Zabet NR, Adryan B (2012). Computational models for large-scale simulations of facilitated diffusion. Mol. Biosyst..

[24] Kolomeisky AB (2011). Physics of protein-DNA interactions: mechanisms of facilitated target search. Phys. Chem. Chem. Phys..

[25] Coppey M, Benichou O, Voituriez R, Moreau M (2004). Kinetics of target site localization of a protein on DNA: a stochastic approach. Biophys. J..

[26] Slutsky M, Mirny LA (2004). Kinetics of protein-DNA interaction: facilitated target location in sequence-dependent potential. Biophys. J..

[27] Sokolov IM, Metzler R, Pant K, Williams MC (2005). Target search of N sliding proteins on a DNA. Biophys. J..

[28] Klenin KV, Merlitz H, Langowski J, Wu CX (2006). Facilitated diffusion of DNA-binding proteins. Phys. Rev. Lett..

[29] Hu T, Grosberg AY, Shklovskii BI (2006). How proteins search for their specific sites on DNA: the role of DNA conformation. Biophys. J..

[30] Benichou O, Loverdo C, Moreau M, Voituriez R (2008). Optimizing intermittent reaction paths. Phys. Chem. Chem. Phys..

[31] Li GW, Berg OG, Elf J (2009). Effects of macromolecular crowding and DNA looping on gene regulation kinetics. Nat. Phys..

[32] Lomholt MA, vanden Broek B, Kalisch SMJ, Wuite GJL, Metzler R (2009). Facilitated diffusion with DNA coiling. Proc. Natl Acad. Sci. USA.

[33] Loverdo C, Bénichou O, Voituriez R, Biebricher A, Bonnet I, Desbiolles P (2009). Quantifying hopping and jumping in facilitated diffusion of DNA-binding proteins. Phys. Rev. Lett..

[34] Meroz Y, Eliazar I, Klafter J (2009). Facilitated diffusion in a crowded environment: from kinetics to stochastics. J. Phys. A Math. Theor..

[35] Vukojevic V, Papadopoulos DK, Terenius L, Gehring WJ, Rigler R (2010). Quantitative study of synthetic Hox transcription factor-DNA interactions in live cells. Proc. Natl Acad. Sci. USA.

[36] Benichou O, Chevalier C, Meyer B, Voituriez R (2011). Facilitated diffusion of proteins on chromatin. Phys. Rev. Lett..

[37] Zhou HX (2011). Rapid search for specific sites on DNA through conformational switch of nonspecifically bound proteins. Proc. Natl Acad. Sci. USA.

[38] Zabet NR, Adryan B (2012). GRiP: a computational tool to simulate transcription factor binding in prokaryotes. Bioinformatics.

[39] Zabet NR, Adryan B (2012). A comprehensive computational model of facilitated diffusion in prokaryotes. Bioinformatics.

[40] van Zon JS, Morelli MJ, Tanase-Nicola S, ten Wolde PR (2006). Diffusion of transcription factors can drastically enhance the noise in gene expression. Biophys. J..

[41] Brackley CA, Cates ME, Marenduzzo D (2012). Facilitated diffusion on mobile DNA: configurational traps and sequence heterogeneity. Phys. Rev. Lett..

[42] Foffano G, Marenduzzo D, Orlandini E (2012). Facilitated diffusion on confined DNA. Phys. Rev. E.

[43] Bauer M, Metzler R (2013). *In vivo* facilitated diffusion model. PLoS One.

[44] Zabet NR, Adryan B (2013). The effects of transcription factor competition on gene regulation. Front. Genet..

[45] Rosenfeld N, Young JW, Alon U, Swain PS, Elowitz MB (2005). Gene regulation at the single-cell level. Science.

[46] Zabet NR (2012). System size reduction in stochastic simulations of the facilitated diffusion mechanism. BMC Syst. Biol..

[47] Stewart WJ (1994). Introduction to the Numerical Solution of Markov Chains.

[48] Gillespie DT (1976). A general method for numerically simulating the stochastic time evolution of coupled chemical reactions. J. Comput. Phys..

[49] Gillespie DT (1977). Exact stochastic simulation of coupled chemical reactions. J. Phys. Chem..

[50] Gillespie DT (2000). The chemical Langevin equation. J. Chem. Phys..

[51] Kolesov G, Wunderlich Z, Laikova ON, Gelfand MS, Mirny LA (2007). How gene order is influenced by the biophysics of transcription regulation. Proc. Natl Acad. Sci. USA.

[52] Brackley CA, Cates ME, Marenduzzo D (2013). Intracellular facilitated diffusion: searchers, crowders, and blockers. Phys. Rev. Lett..

[53] Wunderlich Z, Mirny LA (2008). Spatial effects on the speed and reliability of protein-DNA search. Nucleic Acids Res..

[54] Gerland U, Moroz JD, Hwa T (2002). Physical constraints and functional characteristics of transcription factor-DNA interactions. Proc. Natl Acad. Sci. USA.

[55] Fairall L, Rhodes D, Klug A (1986). Mapping of the sites of protection on a 5 S RNA gene by the Xenopus transcription factor IIIA. A model for the interaction. J. Mol. Biol..

[56] Ruusala T, Crothers DM (1992). Sliding and intermolecular transfer of the lac repressor: kinetic perturbation of a reaction intermediate by a distant DNA sequence. Proc. Natl Acad. Sci. USA.

[57] Sharon E, Kalma Y, Sharp A, Raveh-Sadka T, Levo M, Zeevi D, Keren L, Yakhini Z, Weinberger A, Segal E (2012). Inferring gene regulatory logic from high-throughput measurements of thousands of systematically designed promoters. Nat. Biotechnol..

[58] Gama-Castro S, Salgado H, Peralta-Gil M, Santos-Zavaleta A, Muiz-Rascado L, Solano-Lira H, Jimenez-Jacinto V, Weiss V, Garca-Sotelo JS, Lpez-Fuentes A (2011). RegulonDB version 7.0: transcriptional regulation of *Escherichia coli* K-12 integrated within genetic sensory response units (Gensor Units). Nucleic Acids Res..

[59] Hartigan JA, Hartigan PM (1985). The dip test of unimodality. Ann. Stat..

[60] Maechler M (2012). Diptest: Hartigan’s dip test statistic for unimodality—corrected code. http://cran.r-project.org/web/packages/diptest/index.html.

[61] Blainey PC, Luo G, Kou SC, Mangel WF, Verdine GL, Bagchi B, Xie XS (2009). Nonspecifically bound proteins spin while diffusing along DNA. Nat. Struct. Mol. Biol..

[62] Wang S, Elf J, Hellander S, Lotstedt P (2012). Stochastic Reaction-Diffusion Processes with Embedded Lower Dimensional Structures.

[63] He X, Duque TS, Sinha S (2012). Evolutionary origins of transcription factor binding site clusters. Mol. Biol. Evol..

[64] Lusk RW, Eisen MB (2010). Evolutionary mirages: selection on binding site composition creates the illusion of conserved grammars in Drosophila enhancers. PLoS Genet..

[65] Nourmohammad A, Lässig M (2011). Formation of regulatory modules by local sequence duplication. PLoS Comput. Biol..

[66] Lu P, Vogel C, Wang R, Yao X, Marcotte EM (2007). Absolute protein expression profiling estimates the relative contributions of transcriptional and translational regulation. Nat. Biotechnol..

[67] CoxIII RS, Surette MG, Elowitz MB (2007). Programming gene expression with combinatorial promoters. Mol. Syst. Biol..

[68] Dadiani M, van Dijk D, Segal B, Field Y, Ben-Artzi G, Raveh-Sadka T, Levo M, Kaplow I, Weinberger A, Segal E (2013). Two DNA-encoded strategies for increasing expression with opposing effects on promoter dynamics and transcriptional noise. Genome Res..

[69] Khoueiry P, Rothbcher U, Ohtsuka Y, Daian F, Frangulian E, Roure A, Dubchak I, Lemaire P (2010). A cis-regulatory signature in ascidians and flies, independent of transcription factor binding sites. Curr. Biol..

[70] Janga SC, Collado-Vides J, Babu MM (2008). Transcriptional regulation constrains the organization of genes on eukaryotic chromosomes. Proc. Natl Acad. Sci. USA.

[71] Shen-Orr SS, Milo R, Mangan S, Alon U (2002). Network motifs in the transcriptional regulation network of *Escherichia coli*. Nat. Genet..

[72] Zabet NR, Chu DF (2010). Computational limits to binary genes. J. R. Soc. Interface.

[73] He X, Chen CC, Hong F, Fang F, Sinha S, Ng HH, Zhong S (2009). A biophysical model for analysis of transcription factor interaction and binding site arrangement from genome-wide binding data. PLoS One.

[74] Cheng Q, Kazemian M, Pham H, Blatti C, Celniker SE, Wolfe SA, Brodsky MH, Sinha S (2013). Computational identification of diverse mechanisms underlying transcription factor-DNA occupancy. PLoS Genet..

