# Supplementary material for: Physical constraints determine the logic of bacterial promoter architectures
Source: Nucleic Acids Res. 2014 Jan 28;42(7):4196–207. doi: 10.1093/nar/gku078 (PMC3985651; doi:10.1093/nar/gku078)
Supplement: Supplementary Data [file supp_42_7_4196__index.html]

Physical constraints determine the logic of bacterial promoter architectures — Supplementary Data 

# Physical constraints determine the logic of bacterial promoter architectures

## Supplementary Data

files

**Files in this Data Supplement:**

- Supplementary Data - pdf file
