# Supplementary material for: Physical constraints determine the logic of bacterial promoter architectures
Source: Nucleic Acids Res. 2014 Jan 28;42(7):4196–207. doi: 10.1093/nar/gku078 (PMC3985651; doi:10.1093/nar/gku078)
Supplement: Supplementary Data [file supp_gku078_promoter_architecture_supp.pdf]

SUPPLEMENTARY DATA to:
Physical constraints determine the logic of bacterial promoter architectures

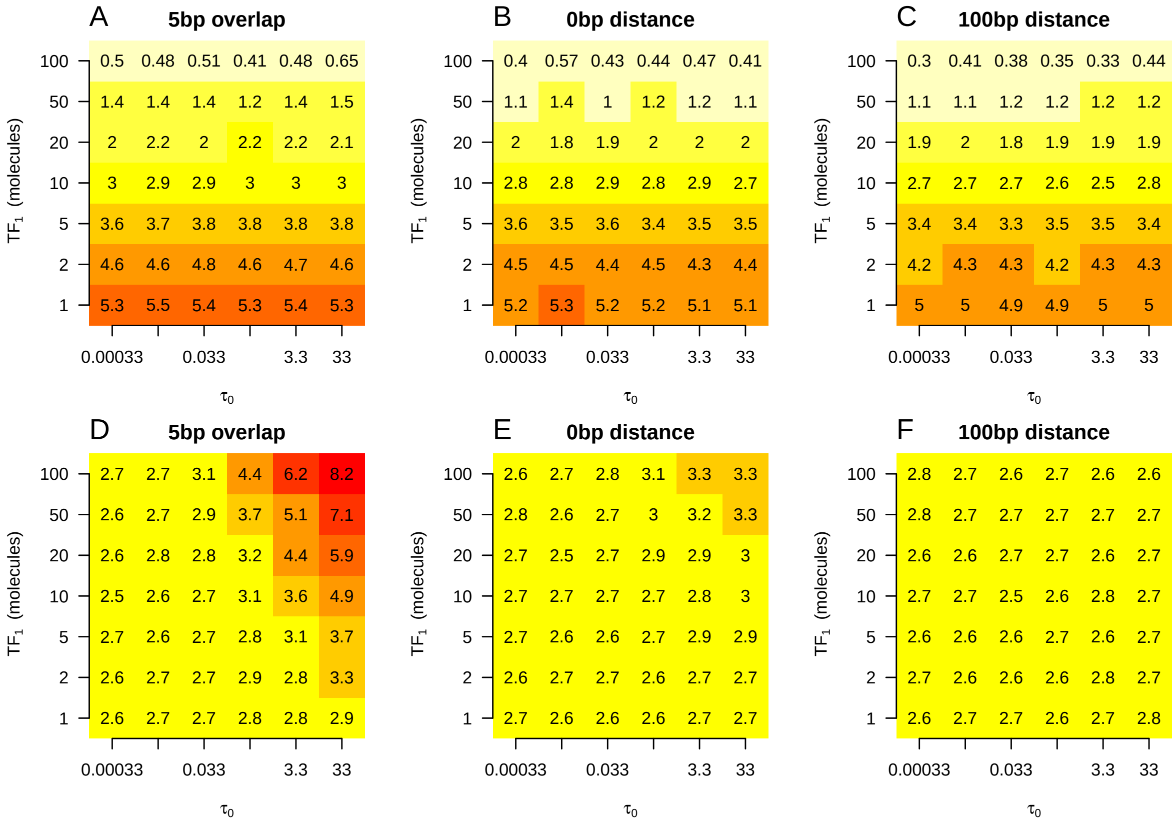

Figure S1: *TF arrival time in fastGRiP for asymmetric system.* We simulated the effects of co-localisation of binding sites for two different types of TFs, which we will refer to as TF_1 and TF_2. For (A) and (D) switches (5 bp overlap); (B) and (E) barriers (0 *bp* distance between sites) and (C) and (F) far apart binding sites (100 *bp* distance between sites). We varied the binding affinity parameter (τ_0) and abundance of TF_1 and kept the parameters of TF_2 constant at $\tau_0 = 0.33$ and 10 molecules. $(A-C)$ show the natural log of the arrival time (the time it takes for TF_1 to first reach its binding site in ln(seconds)) for TF_1 . $(D-F)$ show the natural log of the arrival time for TF_2. These values represent the average ln(arrival times) across 400 simulations. $(A-C)$ We see that in all cases (switch, barrier, far away), TF_1 binds faster at higher abundances, while the binding affinity has no effect on the arrival time of TF_1. We see that binding of TF_1 is slightly slower in switches, and to a lesser extent in barriers, compared to the case of all binding sites being far apart. As we see in (F), changing the parameters of TF_1 have no effect on the binding of TF_2 when the binding sites are far apart. This supposition of positional independence is a key component of most thermodynamical models. However, we show that in the case of switches and barriers, increasing the abundance or binding affinity of TF_1 increases the arrival time for TF_2.

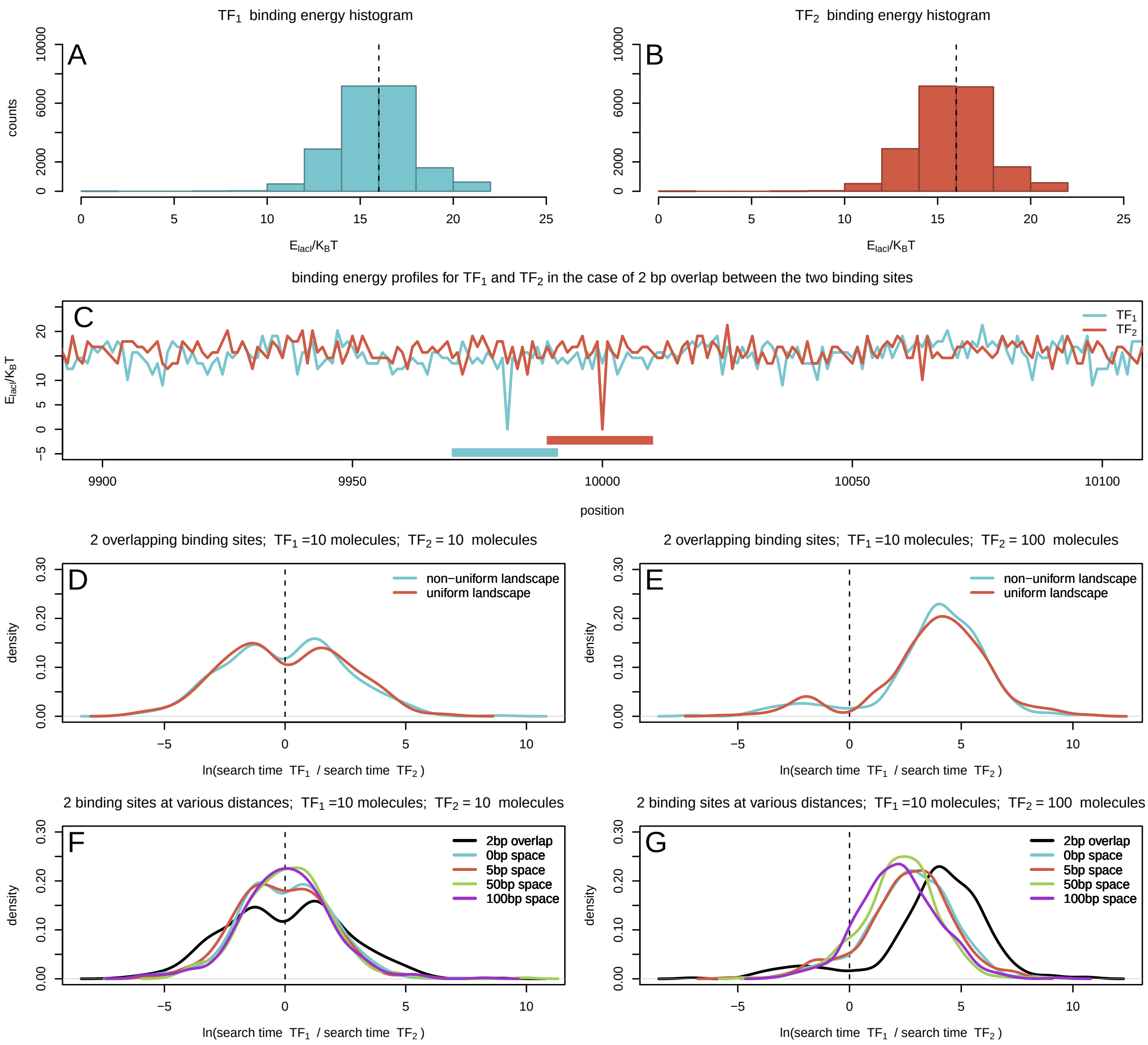

Figure S2: *Facilitated diffusion on a non-uniform landscape.* For the non-uniform landscape, we considered the case of a random 20 Kbp DNA sequence and the two binding sites are located in the middle of the sequence; see (C). The full list of parameters for the non-uniform landscape is listed in Table S4. (A) and (B) Binding energy histogram for the non-uniform landscape for TF_1 (A) and TF_2 (B). The mean binding energy is 16 K_BT, which is the same as in the case of uniform landscape. (C) We also plotted the binding energy profile for the non-uniform landscape case within the region 9900..10100 of the randomly generated landscape. (D) and (E)*Ratio of TF arrival times for switches* . We performed a set of $X = 200$ simulations for each set of parameters in the case of the uniform landscape and $X = 400$ in the case of non-uniform landscape. The two sites for the two TFs overlap by 5 bp in the case of the uniform landscape and 1 bp in the case of the non-uniform landscape. The two TFs have the same abundance ($TF_1 = TF_2 = 10$) in (D) and different abundances ($TF_1 = 10$ and $TF_2 = 100$) in (E). (D) For a non-uniform landscape, we found that the TF_2 gets first to its target site in 51.25% while TF_1 in 48.75% of the cases when both TFs have the same set of parameters, thus, creating an slightly unbalanced system. When computing the waiting time within one sliding length of the target site, We found that TF_2 is on average 4.2% faster in the 1D movement compared to TF_1. Thus, in a symmetric system, the TF that moves faster within half of sliding length from the target site can reach its target site first. This result contradicts the prediction that slowing down the TF near the target site (also known as the funnel effect) reduces the search time (41, 75, 76) but, in those studies, the authors considered a biased random walk and did not consider multiple sites and multiple DNA binding proteins. (F) and (G) *Ratio of TF arrival times for switches and barriers.* We performed a set of $X = 400$ simulations for each set of parameters. The two sites for the two TFs overlap by 1 bp or are separated by: 0 bp, 5 bp, 50 bp and 100 bp. (G) When TF_2 has a higher abundance than TF_1 ($TF_1 = 10$ and $TF_2 = 100$), then decreasing the distance between the two sites accentuates the difference between the arrival time at the two sites. These results are similar with the case of uniform landscape, except that the distributions are not as separated as in the case of uniform landscape.

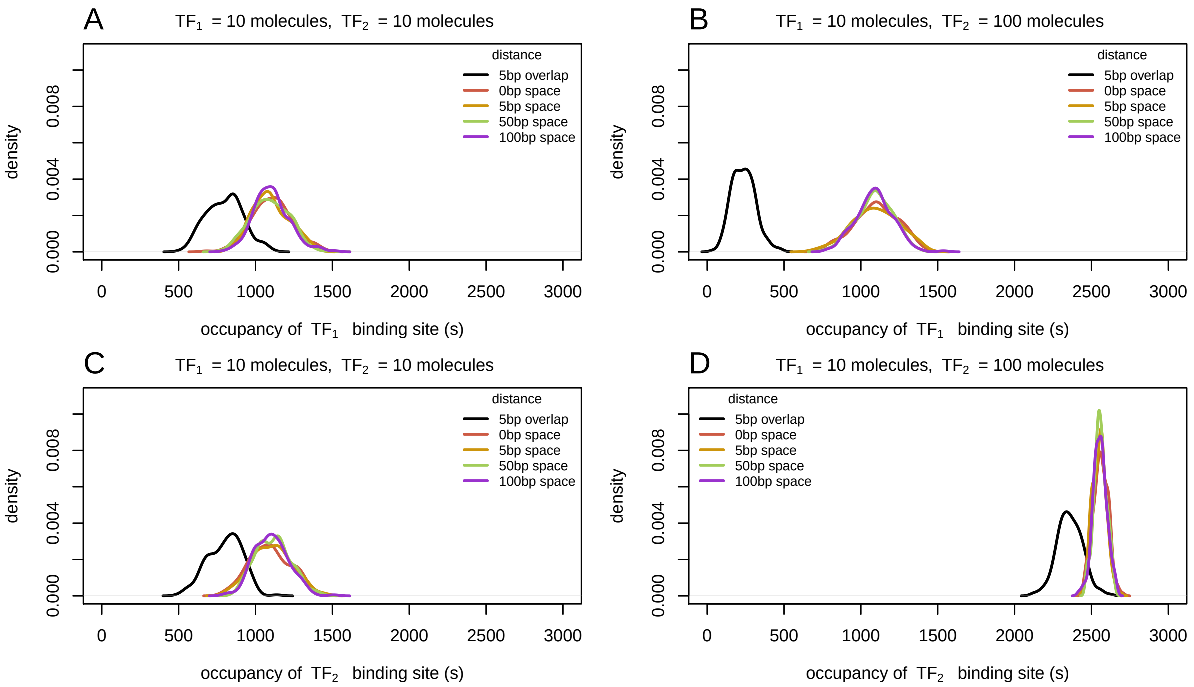

Figure S3: *Occupancy of switches and barriers in GRiP.* Here we simulated the switch and barrier scenarios with various distances between the binding sites (for switch, 5 *bp* overlap and, for barriers, $\in \{0, 5, 50, 100\}$ *bp* space between the two binding sites). We measured the occupancy (the total amount of time a binding site is bound during a period of time) across an *E. coli* cell cycle (3000 seconds) when (A) and (C) $TF_1 = TF_2 = 10\, molecules$ and (B) and (D) $TF_1 = 10\, molecules$ and $TF_2 = 100\, molecules$. Binding sites in the switch configuration have significantly less occupancy than binding sites that are far apart and higher variability in occupancy. On the other hand, barriers do not seem to have a significant effect on occupancy. (A) and (B) the occupancy for TF_1. (C) and (D) the occupancy for TF_2.

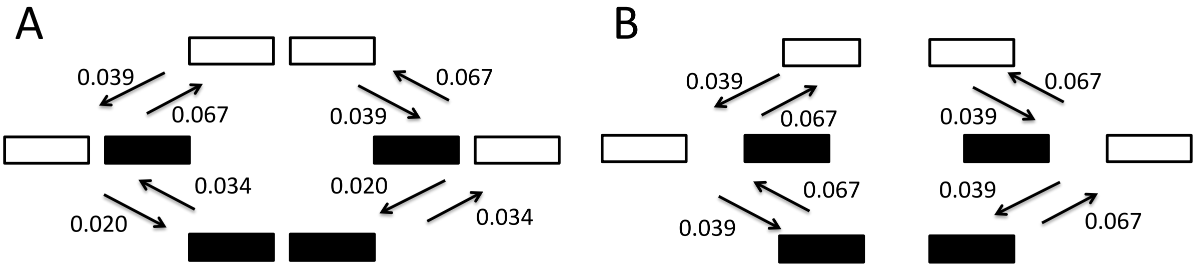

Figure S4: *fastGRiP transition diagrams for barriers and far apart binding sites.* One of the reasons why overall TF occupancy is not significantly affected by the *barrier effect* is that the both the rate of binding and unbinding events is reduced in the barrier case. Here we show the Markov Chains used in fastGRiP that describe a barrier (A) and two far apart binding sites (B). In fastGRiP, the states represent specific configurations (white boxes are unbound sites and black boxes are bound sites) and transitions represent specific binding or unbinding reactions. Along the arrows that represent the state transitions, we show the propensities of binding and unbinding events, as calculated by fastGRiP for (A) barriers and (B) far apart binding sites . The expected reaction time is inversely proportional to the reaction propensity. We see that the barriers case has lower or equal reaction propensities for all TF binding and TF unbinding reactions compared to the case of far away binding sites. Therefore, there will be less frequent binding and unbinding events if the TF binding sites are closely spaced. Even though we saw in that the overall occupancy (the total time a TF is bound across a cell cycle) is not significantly affected by barriers (Figure S3), we see that barriers influence the TF binding kinetics.

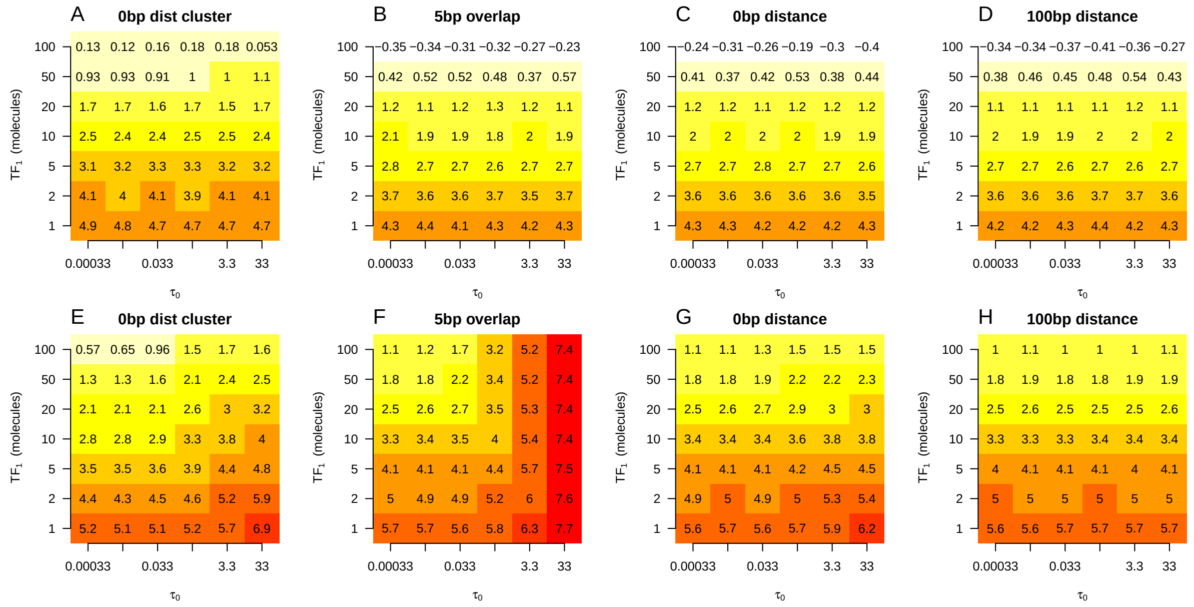

Figure S5: *TF arrival time in fastGRiP for symmetric system.* We change the abundance and binding affinity of *both* TFs in the system symmetrically. In other words, we simulate a pair of co-localised binding sites, changing the binding affinities and abundances of both TFs in the system. We observe the arrival time (in ln(seconds)) for the first of the two TFs, $ln(min(search\ time\ TF_1, search\ time\ TF_2))$, in $(A - D)$ and the last of the two TFs, $ln(max(search\ time\ TF_1, search\ time\ TF_2))$, in $(E - H)$. We ran this experiment on (A) and (E) clusters (0 bp distance between sites); (B) and (F) switches (5 bp overlap between sites); (C) and (G) barriers (0 bp distance between sites) and (D) and (H) far away binding sites (100 bp distance between sites). As before, the values represent the averages across 400 simulations. We see that TF abundance influences the time of binding of the first arriving TF, but binding affinity does not affect the first TF's arrival time. The first arriving TF in a cluster is slower than the switch, barrier, or far away cases, because a neighbouring binding site in a cluster can act like a trap (because the TF will bind to the first TF binding site it bumps into). As shown in (F) and (G), in the switch case, and to a lesser extent in the barrier case, increasing binding affinity slows the binding of the latest arriving TF. Most importantly, in (E) we can see the dual nature of cluster: at high levels of binding affinity and low concentrations, the binding of the second TF is slowed. On the other hand, at low binding affinities and high concentrations, the second TF will bind faster in the case of a cluster than it would in the case of the two binding sites being far apart, because the TF can slide back and forth between the two binding sites.

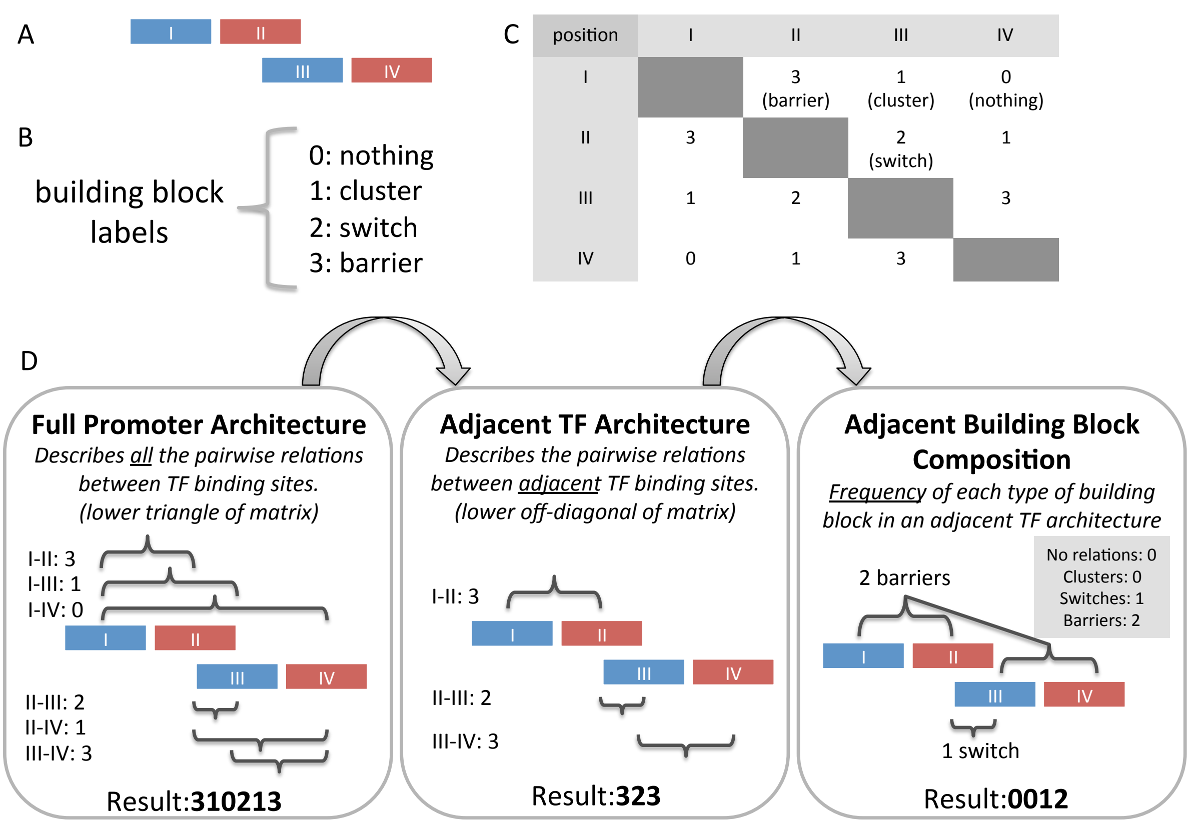

Figure S6: *Promoter architecture notation.* It is extremely useful to classify promoter organisations by their composition of switches, barriers, and clusters in an unambiguous and enumerable way. Here we present a versatile scheme for enumerating promoter architectures. (A) shows an example promoter architecture, which we will use as an example to demonstrate the algorithm we use to categorise them, comprising binding sites I to IV. (B) shows our mapping of numerals to building blocks, which we can use to label a particular pairwise interaction. (C) shows all of the pairwise interactions between TFs for our example case. For instance, as we see that binding site I and binding site II form a barrier, we put the number 3 in the corresponding location in the table. In (D), we describe how we can transform this table into numerical labels for the promoter architecture. First, we define the *full promoter architecture label* to describe all pairwise interactions between TFs and their binding sites. We show the specific order of comparisons we make in order to have a consistent labeling scheme. Note that this corresponds to the lower triangular portion of the table in (B), read column by column. Next, we define the *adjacent TF architecture label* as the description of all pairwise interactions between TFs that are immediately adjacent to one another. This corresponds to the upper or lower off-diagonal of the table. In this example, we see clusters are part of the full promoter architecture, but not part of the adjacent TF architecture, because binding sites I and III nor binding sites II and IV are immediately adjacent to one another. These relationships between adjacent TFs often contribute the most to the behavior of the promoter architecture. Finally, we refer to the counts of each type of building block as the *adjacent building block composition label.* This label represents the frequency of observing switches, barriers, and clusters among adjacent pairs of binding sites. Our enumeration strategy is particularly useful because: it defines a way of easily label promoter architectures by its building blocks and also defines a hierarchy of promoter organisations (from promoter architecture to adjacent binding sites architecture to building block composition). This helps group promoters based on their architectures and look at the distribution of architectures across the genome more effectively. (Note that if the promoter architecture was drawn in the opposite orientation, then the table could change, so to keep consistency, we always take the label that has the highest value.)

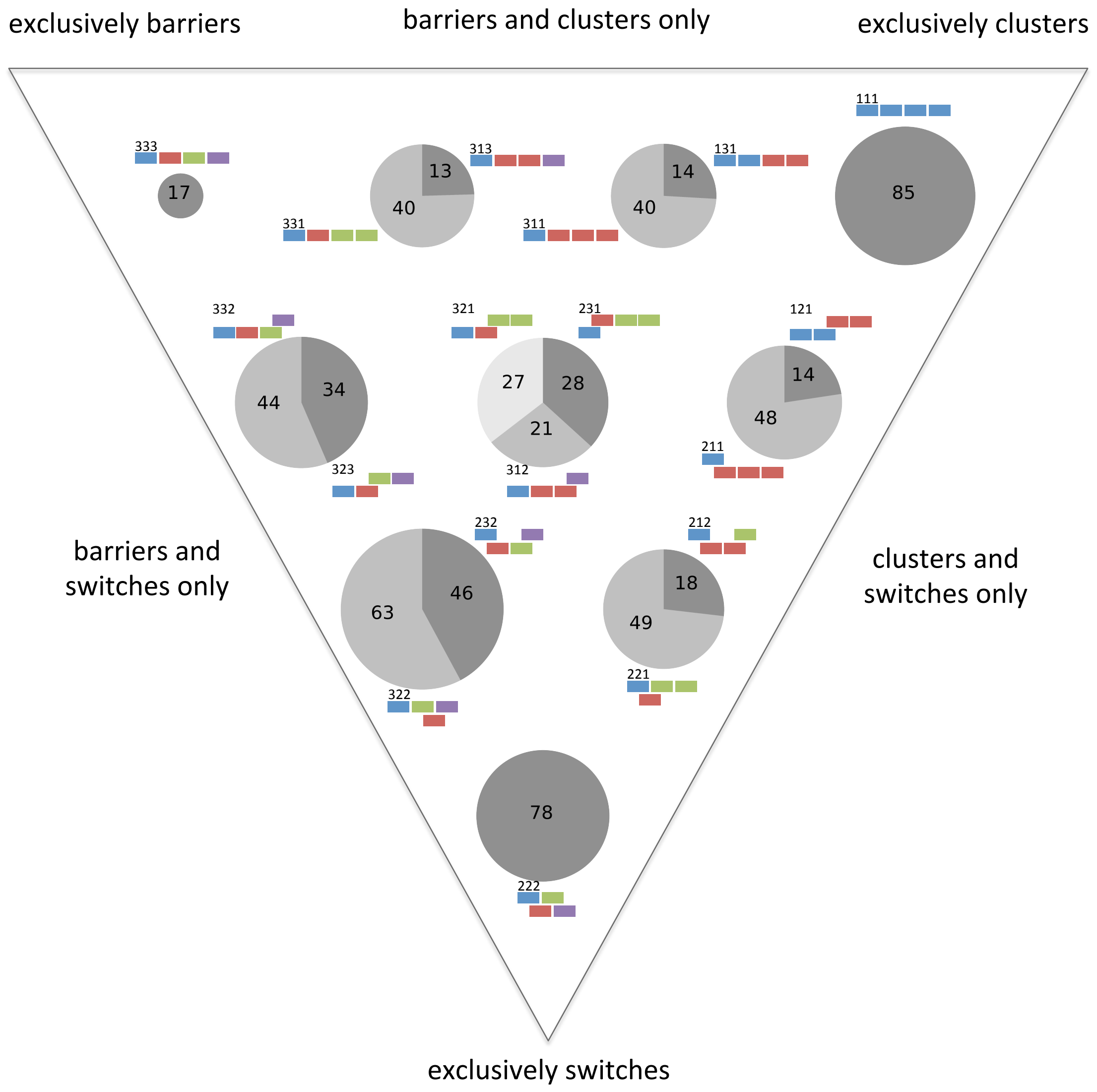

Figure S7: *Distribution of sets of 4 binding sites in the* E. coli *genome.* We show the distribution of all sets of four binding sites < 100 *bp* apart. Each circle represents a different building block composition, and the proximity of each circle to each corner corresponds with how many barriers, switches, and clusters are present. For instance, the circles in the corners represent architectures that consist of only one type of building block (such as, only barriers). The circle in the center represents architectures with one of each type of building block (one barrier, one cluster and one switch). The size of each circle represents the number of times we observe that building block composition in the *E.coli* genome. Within each circle is a pie chart that shows the relative frequencies of each adjacent TF architecture. The numbers inside the pie chart correspond to the number of times that adjacent TF architecture was found in the *E.coli* genome. Each of the architectures are also drawn, and labelled with their associated adjacent TF architecture label.

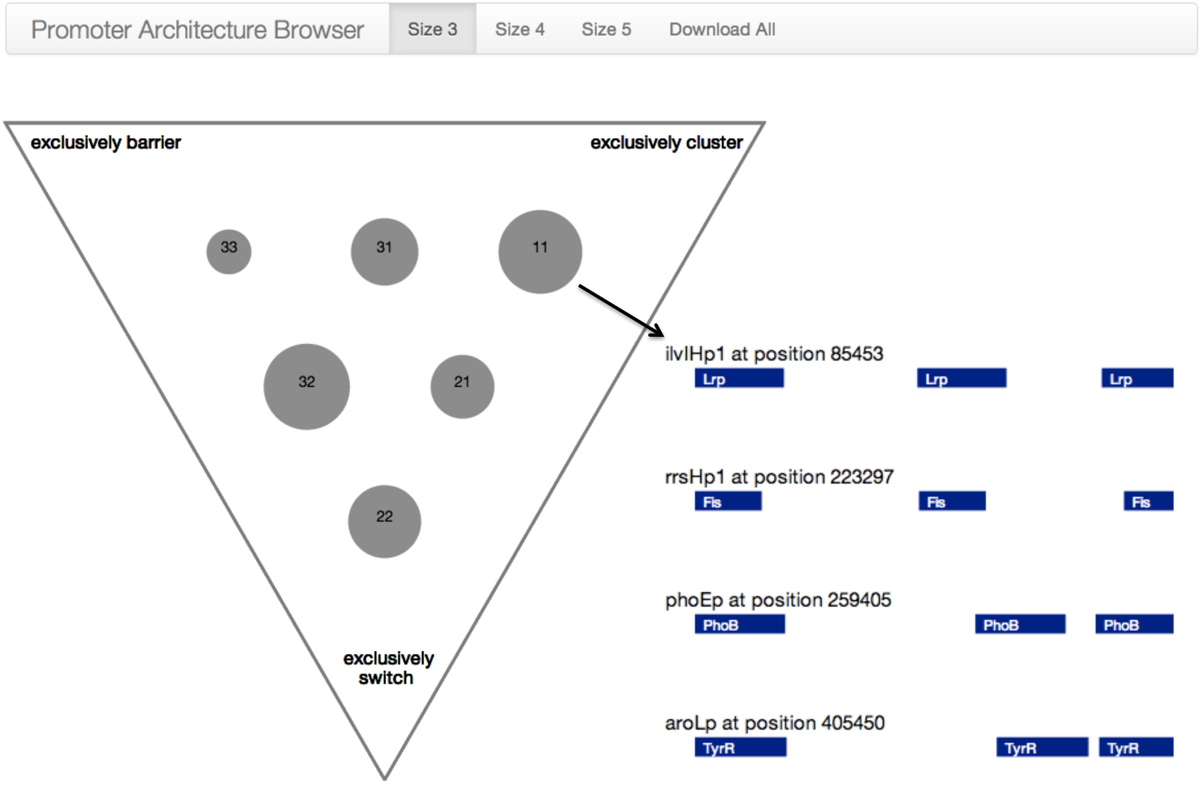

Figure S8: *Webtool for browsing binding site architectures in the* E. coli *genome.* We have classified promoter architectures of every set of 3, 4, and 5 TF binding sites that are less than 100 bp apart from one another. To easily browse the *E.coli* architectures, we have created a website (http://logic.sysbiol.cam.ac.uk/fgrip/db) that allows the user to click of each part of the pie chart and scroll through the corresponding *E.coli* promoters.

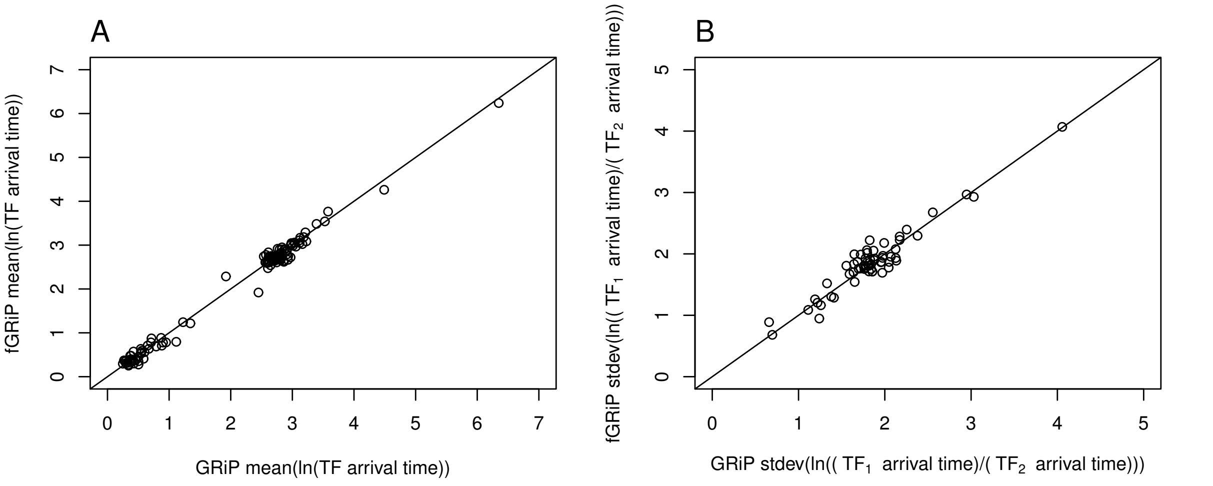

Figure S9: *Comparison between GRiP and fastGRiP.* Here we compare the mean of the log of the arrival times (A) and the standard deviation of the log of the arrival ratios (B) between GRiP, the fully stochastic simulation, and fastGRiP, the semi-analytic approximation. Each point represents one building block experiment (a switch, barrier, or cluster) under a specific parameter set: a TF abundance of 10 or 100, a binding affinity t_0 value of 0.033, 0.33, or 3.3, and a distance between binding sites of -5 (overlap), 0, 5, 50, or 100. (A) shows that the values for the arrival times are consistent between the simulations, with $R = 0.995$. (B) shoes that the relative arrival times are consistent between the simulations, with $R = 0.954$.

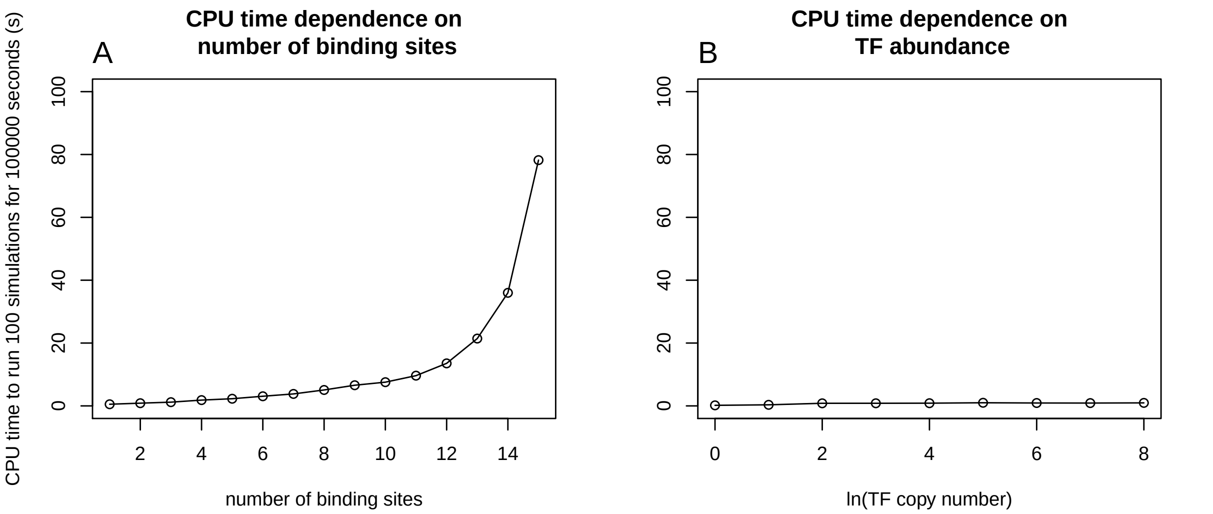

Figure S10: *Time required to simulate* 10^5 *s with fastGRiP.* The simulations were performed on a MacBook Air 1.8 GHz Intel i5 CPU with 4GB memory running Mac OSX 10.7. (*A*) The dependence of the the CPU time on the number of binding sites, in the case of 1000 molecules. fastGRiP becomes significantly slow for high number of binding sites. However, for usual number of binding sites in bacterial promoters, fastGRiP is fast. (*B*) The dependence of CPU time on the number of molecules, in the case of 2 binding sites. Practically, fastGRiP simulation time is not affected by the number of molecules. This is opposite to GRiP where there is linear dependence with the length of the DNA (and, thus, with the number of binding sites) and exponential dependence on the number of molecules.

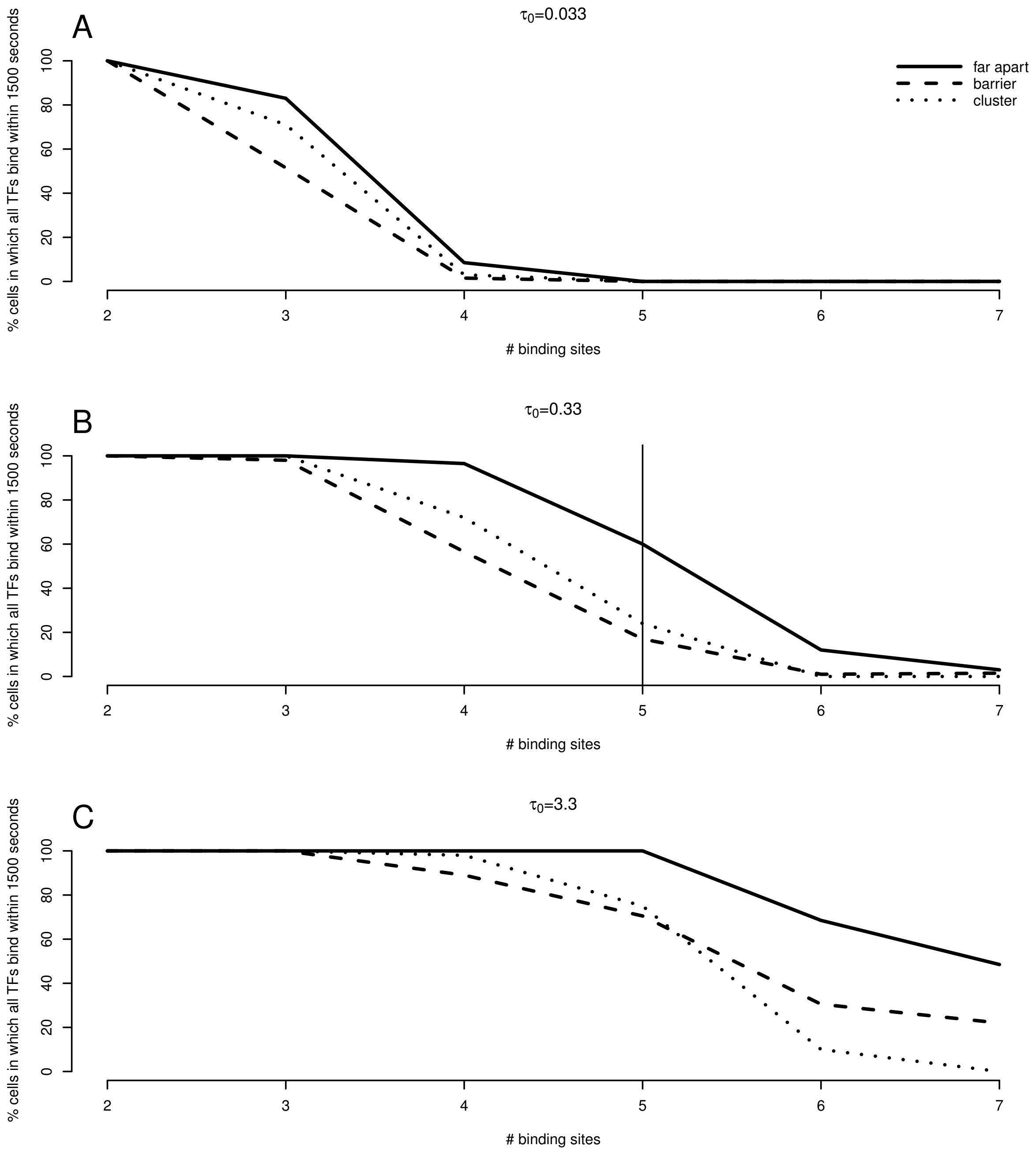

Figure S11: *Influence of promoter architecture on formation of AND configuration.* Here we show the proportion of cells (simulations) in which the AND configuration (all TFs bound at once) is reached before half a cell cycle (1500 seconds), dependent on the number of binding sites and the organisation of the promoter (TFs far apart and therefore unaffected by facilitated diffusion– solid line, TFs 0bp apart in a barrier organisation– dashed line and TFs 0bp apart in a cluster organisation– dotted line). Each graph shows the results at a different level of binding affinity ($\tau_0 = 0.033$ in (A), $\tau_0 = 0.33$ in (B) and $\tau_0 = 3.3$ in (C)), but a constant TF abundance of 10 molecules. As expected, in all cases the higher the complexity of the promoter, the less likely it is for all TFs to find their binding sites within half a cell cycle; however, when the TFs are far apart from one another, a higher proportion of cells would reach the AND configuration within half a cell cycle than in the case of barriers or clusters. For example, as indicated by the horizintal line in (B), in the case of a τ_0 of 0.33 and 5 binding sites, the number of cells that reach the AND configuration within half a cell cycle is reduced by more than half between the far away case and the barrier/cluster cases (from 60% to 24%). Notice that the higher the binding affinity between the TFs and the DNA, the larger the effect of closely packing TF binding sites on the formation of the AND configuration. At lower binding affinities, the relative effect is smaller, but one begins to see an effect when there are fewer adjacent binding sites.

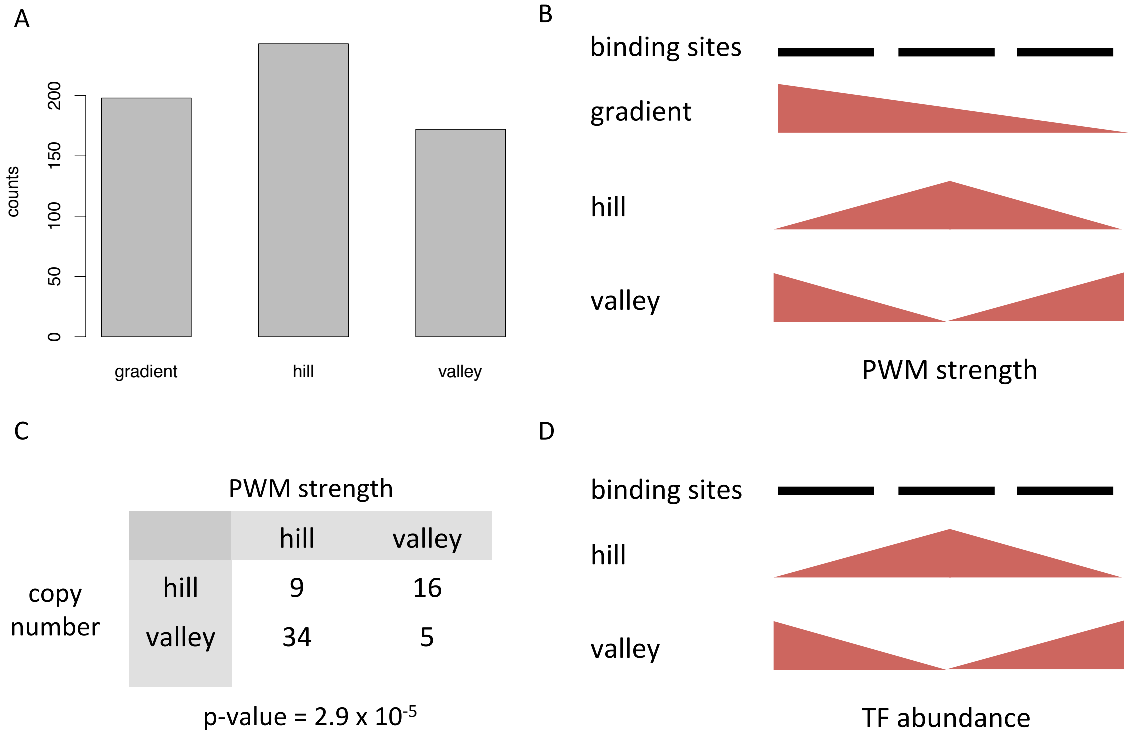

Figure S12: *Hill and valleys in E. coli genome.* When three binding sites are closely spaced, the relative binding affinities and abundances of the outer binding sites compared to the central binding site influences the rate at which all three binding sites can be bound at once (*AND configuration*). We define a *hill* to be a configuration in which the central binding site has a higher binding affinity (B) or abundance (D) and a *valley* to be a configuration in which the central binding site has the lowest binding affinity (B) or abundance (D). Other configurations are classified as *gradients*. We categorized binding sites triplets in *E. coli strain K-12* that were $< 50bp$ apart from one another as hills, valleys, or gradients in terms of PWM scores (A) and abundances (C). (A) The distribution of hills, valleys, and gradients in terms of PWM scores is significantly different from the expected distribution (p-value= 0.0018, chi-squared test), with an enrichment for hills. When we calculated hills and valleys in terms of abundances of TFs (C), we only considered triplets that had two or more TFs that had high enough abundances such that their concentrations could be measured by APEX (66). Hills and valleys in terms of binding affinity and concentration appear to be anti-correlated and the results vary significantly from the null distribution (p-value= 2.9×10^{-5}, Fisher exact test). Triplets that are hills in terms of binding affinity and valleys in terms of concentration are best suited for having multiple TFs bound at once, and this seems to be the case within the *E.coli* genome.

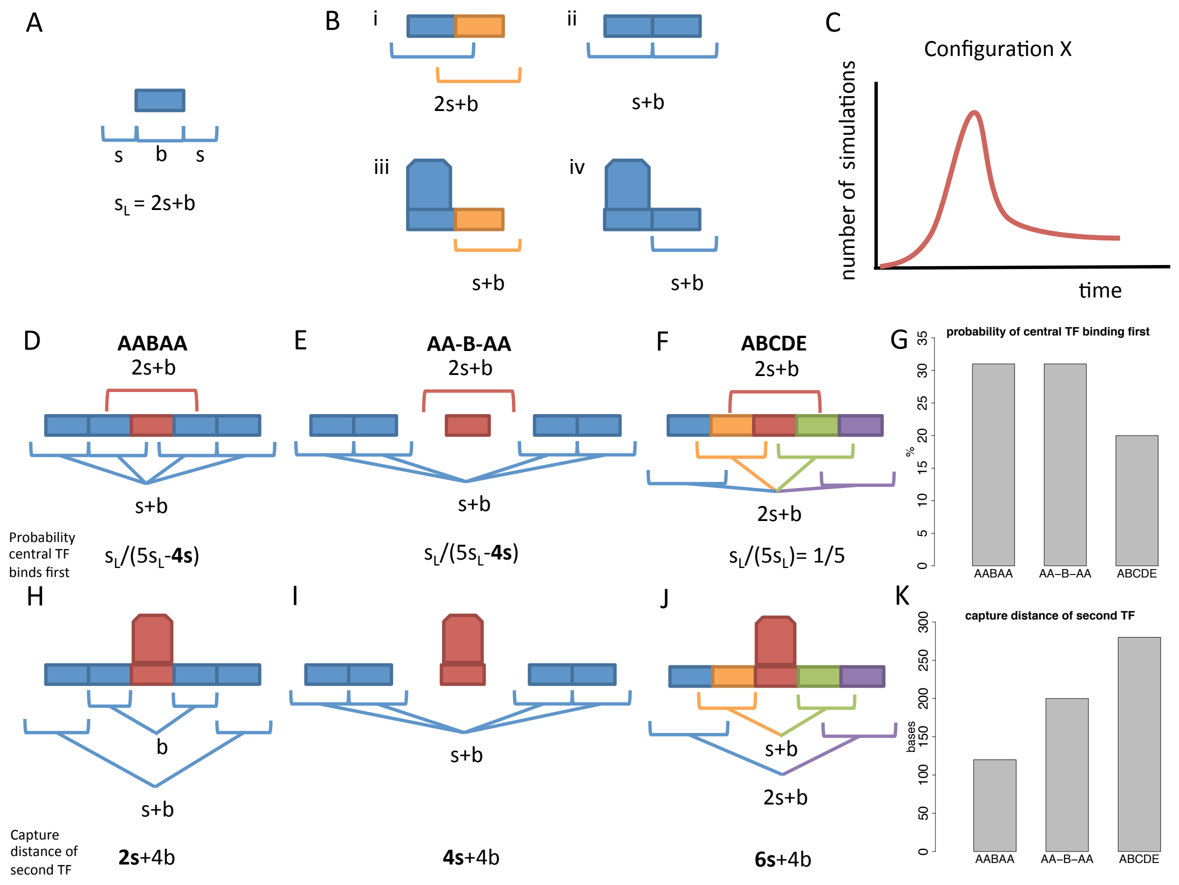

Figure S13: *Mechanism underlying impulse behavior.* In (A) the sliding length (s_L) is defined as the length of the DNA segment where a TF will almost certainly find its binding site by 1D random walk. For the purpose of this figure, we can describe s_L as the sum of the length of the binding site b and a region on either side of the binding site, s bases on each side. (B) Here we illustrate how the sliding length varies in the case of barriers (i, iii) and clusters (ii, iv), and when the adjacent site is unbound (i, ii) or bound (iii, iv). Subfigures (iii, iv) re-iterate the barrier effect, while subfigure (ii) illustrates that a TF will bind to the first site it reaches, and therefore *unbound* binding sites in a cluster act like barriers. (C) A configuration is defined as the combination of TFs that are bound to the DNA at a given time point (such as the case of TF_B being bound, but no TF_A being bound in an AABAA architecture). We define an impulse behavior as a short period of time in which there is a higher probability of a certain configuration occurring than observed at equilibrium Note that two of the phenomenon that influence this include 1) how often the central TF binds first, which we will describe further for the AABAA, AA-B-AA, and ABCDE cases in figures (D-G) and 2) the time delay before additional TFs bind, which we explore further in figures (H-K). In (D-F) we illustrate the sliding widths for each binding site in the case of empty DNA. Figures (D-F) show how there is a relative higher probability of the central TF becoming bound in the AABAA and AA-B-AA cases (D, E) compared to the ABCDE case (F). Using $b = 10$ and $s = 40$ (biologically relevant parameters), we show how the probability of the central TF binding first differs between the three cases. In (H-K), we illustrate the total sliding widths for each binding site when only the central TF is bound. The AABAA configuration has the lowest total sliding width (H) compared to the AA-B-AA and ABCDE (I, J) cases, which implies that the AABAA configuration would result in the longest time delay before a second TF becomes bound. Figure (K) compares total capture distance (region in which a TF will likely bind to one of the sites if it binds there) in each of the three cases with the same parameters as in (G). This is one way of interpreting the underlying mechanism that results in AABAA having the largest magnitude of an impulse, while AA-B-AA has a lower magnitude of an impulse and ABCDE barely has an impulse at all.

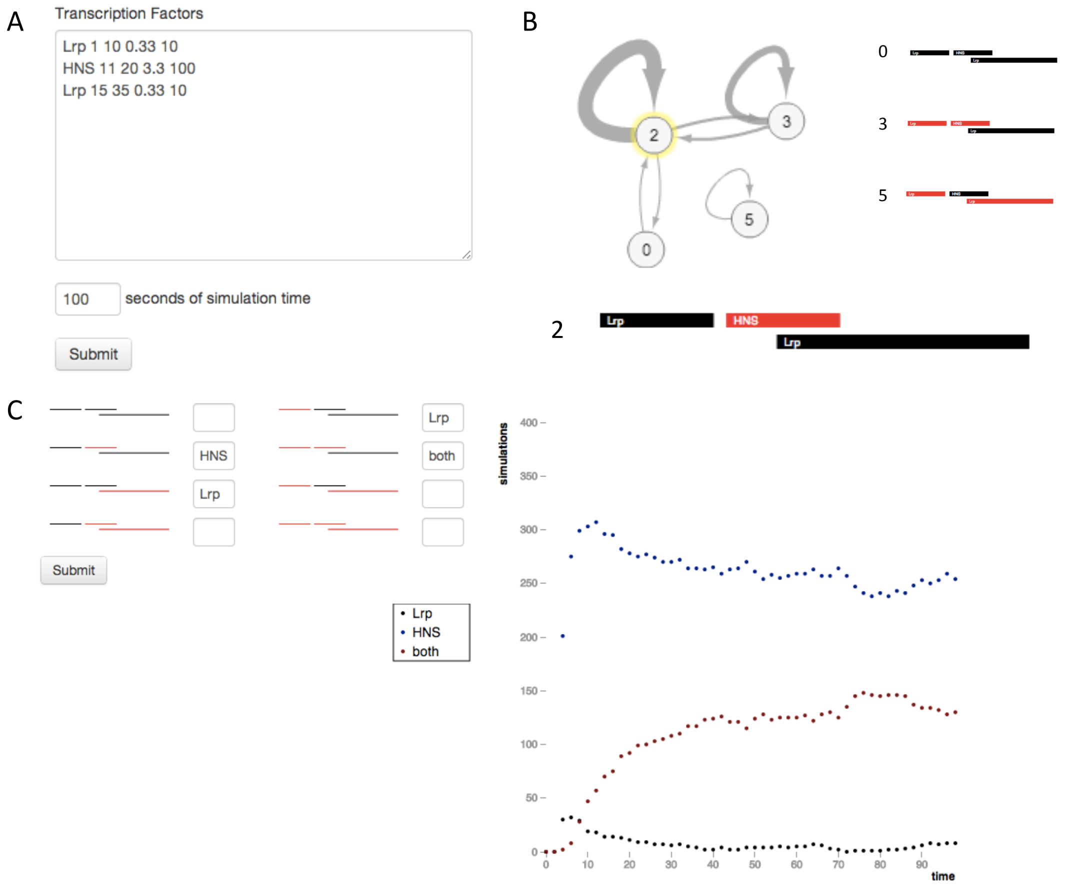

Figure S14: *fastGRiP website user interface.* Here we show screen captures from an exemplary promoter (http://logic.sysbiol.cam.ac.uk/fgrip/). In (A) we see the input screen, filled with demo data. The TF names, start and end positions of the binding sites, the τ_0 binding affinity parameter, and TF abundance must be specified, along with the number of seconds of the *E.coli* life cycle for the simulation to run. In (B), we see a network output for fastGRiP, where each node represents a specific promoter configuration (for instance, configuration 3, has the first two TFs bound). The thickness of the arrows indicates the frequency of a particular configuration-to-configuration transition across 5 seconds. For instance, if the cell is in configuration 2 (HNS only bound), then it will likely continue to be in configuration 2 after 5 seconds. This network helps illustrate the dynamics of how promoter configurations change over time. When one clicks on a node, it gets highlighted (as shown for config 2), and the configuration map is illustrated. In (C), we show another graphical output of fastGRiP. All the possible configurations are illustrated to the left, with a place in which the user can specify labels for the configurations of interest. After clicking the submit button, a scatterplot will graph the frequency of those configurations over time. If two configs have the same label, the scatterplot will display the sum of the frequency of their configurations.

parameter	description
M	the length of the DNA; $M \approx 4.6$ Mbp (77).
f	the proportion of time a molecules spends on the DNA; $f \approx 0.9$ (18).
t_R	residence time, the amount of time a TF performs a one dimensional random walk on the DNA, before it unbinds; $t_R = 5$ ms (18).
k^{dissoc}	the dissociation rate constant between a TF molecule and the DNA; $k_x^{\text{dissoc}} = 1/t_R = 200$ (39).
k_x^{assoc}	the association rate constant for a TF to the DNA; for the full system, $k_x^{\text{assoc}} = 2400$ s^{-1} (39), while for smaller system we used equation 10 in (46).
s_l	sliding length, the average number of positions on the DNA scanned during a one dimensional random walk; $s_l = 90$ bp (18).
P_{unbind}	the probability to unbind from the DNA; $P_{\text{unbind}} = 0.00147$ (39).
P_{left}, P_{right}	the probability to slide left (P_{left}) or right (P_{right}) on the DNA; $P_{\text{left}} = P_{\text{right}} = 0.4994$ (39).
P_{jump}	the probability that the molecule releases into the cytoplasm during an unbinding event, while $(1 - P_{\text{jump}})$ represents the probability that a molecule will rebind fast after a dissociation (hop); $P_{\text{jump}} = 0.1675$ (53).
σ^2_{hop}	the variance of the hop distance, which is Gaussian distributed around unbinding position;$\sigma^2_{\text{hop}} = 1$ bp (53).
d_{jump}	the distance over which a hop becomes a jump; $d_{\text{jump}} = 100$ bp (53).
τ_x^0	the average waiting time for species x when bound specifically; here we used the value of $\tau_x^0 = 0.33$ which was derived in (39) for non-cognate TFs.
ζ	the pseudo-count term for the PWM; $\zeta = 1$ (78).
TF_x^{size}	the number of base pairs covered by a bound TF molecule; $TF_{\text{nc}} \geq 20$ bp (79).
TF_x^{motif}, TF_x^{left} and TF_x^{right}	the number of base pairs covered by the DNA binding domain (TF_x^{motif}), to the left (TF_x^{left}) of the DNA binding domain and to the right (TF_x^{right}) of the DNA binding domain. When we provide the affinity landscape to GRiP (thus, not specifying TF_x^{size}), we use the TF_x^{left} and $TF_x^{\text{right}} = 0$ to denote that a bound molecule covers $TF_x^{\text{left}} + TF_x^{\text{right}} = 0$ base pairs on the DNA.
K_B and T	Boltzmann constant (K_B) and temperature (T).

Table S1: *Nomenclature.* This is a subset of the parameters associated with the facilitated diffusion, which were listed in (39).

parameter	TF_1	TF_2	notation
copy number	10	10	TF_x
motif sequence	C	G	
energetic penalty for mismatch	$16K_BT$	$16K_BT$	ε_x^*
nucleotides covered on left	10 *bp*	10 *bp*	TF_x^{left}
nucleotides covered on right	10 *bp*	10 *bp*	TF_x^{right}
association rate to the DNA	0.86 s^{-1}	0.86 s^{-1}	k_x^{assoc}
unbinding probability	$1.47E-3$	$1.47E-3$	P_x^{unbind}
probability to slide left	0.4992629	0.4992629	P_x^{left}
probability to slide right	0.4992629	0.4992629	P_x^{right}
probability to dissociate completely when unbinding	0.1675	0.1675	P_x^{jump}
time bound at the target site	0.33 s	0.33 s	τ_x^0
the size of a step to left	1 *bp*	1 *bp*	
the size of a step to right	1 *bp*	1 *bp*	
variance of repositioning distance after a hop	1 *bp*	1 *bp*	σ^2_{hop}
the distance over which a hop becomes a jump	100 *bp*	100 *bp*	d_{jump}

Table S2: *TF species default parameters.* We consider the case of two TF species TF_1 and TF_2 and a uniform affinity landscape. This is implemented by assuming that the DNA is a string form only of adenine and at the target site (usually position in the middle of the DNA) we consider that the DNA contains the 1-bp recognition motifs for the two TFs, namely: cytosine for TF_1 and guanine for TF_2. We considered a 20 *Kbp* DNA segment which is smaller compare to the *E.coli*-K12 genome (which is 4.6 *Mbp*) and, thus, we applied the association rate model to adapt the association rate of the small subsystem from $k_x^{\text{assoc}} = 2400\ s^{-1}$ to $k_x^{\text{assoc}} = 0.86$ (46).

parameter	TF_1	TF_2	notation
association rate to the DNA	$1.98E8\ s^{-1}$	$1.98E8\ s^{-1}$	k_x^{assoc}
unbinding probability	1.0	1.0	P_x^{unbind}
probability to slide left	0.0	0.0	P_x^{left}
probability to slide right	1.0	1.0	P_x^{right}
probability to dissociate completely when unbinding	1.0	1.0	P_x^{jump}
time bound at the target site	1336.5 s	1336.5 s	τ_x^0

Table S3: *TF species default parameters for the case when TF molecules perform only 3D diffusion* To match the number of events performed on the DNA as in the case of facilitated diffusion ($1.0E8$ events) we changed the association rate to $1.98E8\ s^{-1}$ and simulated the system for 84600 s. The specific waiting time was increased by 4050 in order to include the average number of 1D events performed during one random walk on the DNA; $\tau_0^* = (s_l^2/2)\tau_0$, where s_l is the sliding length, which we approximated to be 90 *bp*. The rest of the parameters have the values as listed in Table S2.

parameter	TF_1	TF_2	notation
motif sequence	GACTATAGCTTACAAAAAA	CTCTATTATGAGCAACGGT	
energetic penalty for mismatch	$1.122K_BT$	$1.122K_BT$	ε_x^*
nucleotides covered on left	1 *bp*	1 *bp*	TF_x^{left}
nucleotides covered on right	1 *bp*	1 *bp*	TF_x^{right}

Table S4: *TF species default parameters for the non-uniform landscape.* TFs will cover the same number of nucleotides as in the case of a uniform landscape, namely 21 *bp*. For the non-uniform landscape, we considered a random 20 *Kbp* DNA sequence using the *E.coli* K-12 nucleotide composition ($A = 24.6\%$, $T = 24.6\%$, $C = 25.4\%$ and $G = 25.4\%$). In addition, the two TF species display four different parameters compared to the uniform landscape case, namely: the motif sequence, the energetic penalty for a mismatch and covered nucleotides to the left and to the right of the motif. Note that the TF motifs were generated based on the nucleotide compositions of the motifs in *E.coli* ($A = 29.2\%$, $T = 29.9\%$, $C = 20.4\%$ and $G = 20.5\%$). We selected a penalty of $1.122K_BT$ for an energy mismatch, which is within biological plausible parameters ($[1K_BT, 3K_BT]$) (54) and which ensures that the average binding energy is $-16K_BT$ as in the case of the uniform landscape; see Figure S2*(A-C)*. The rest of the parameters have the values as listed in Table S2.

References

75. Weindl, J., Hanus, P., Dawy, Z., Zech, J., Hagenauer, J., and Mueller, J. C. (2007) Modeling DNA-binding of Escherichia coli σ^{70} exhibits a characteristic energy landscape around strong promoters. *Nucleic Acids Res.* **35(20)**, 7003–7010.

76. Weindl, J., Dawy, Z., Hanus, P., Zech, J., and Mueller, J. C. (2009) Modeling promoter search by *E. coli* RNA polymerase: One-dimensional diffusion in a sequence-dependent energy landscape. *J. Theor. Biol.* **259(3)**, 628–634.

77. Riley, M., Abe, T., Arnaud, M. B., Berlyn, M. K., Blattner, F. R., Chaudhuri, R. R., Glasner, J. D., Horiuchi, T., Keseler, I. M., Kosuge, T., Mori, H., Perna, N. T., Plunkett, G., Rudd, K. E., Serres, M. H., Thomas, G. H., Thomson, N. R., Wishart, D., and Wanner, B. L. (2006) *Escherichia coli* K-12: a cooperatively developed annotation snapshot - 2005. *Nucleic Acids Res.* **34(1)**, 1–9.

78. Berg, O. G. and vonHippel, P. H. (1987) Selection of DNA Binding Sites by Regulatory Proteins Statistical-mechanical Theory and Application to Operators and Promoters. *J. Mol. Biol.* **193(4)**, 723–750.

79. Stormo, G. D. and Fields, D. S. (1998) Specificity, free energy and information content in protein-DNA interactions. *Trends Biochem. Sci.* **23(3)**, 109–113.
